# Kinetics of droplet cloaking and wetting ridge growth on lubricated polymer brushes

**DOI:** 10.1140/epje/s10189-026-00607-6

**Published:** 2026-07-20

**Authors:** Antonio Torregrosa Abellán, Enqing Liu, Vincent Siekman, Frieder Mugele, Friederike Schmid, Rodrique G. M. Badr

**Affiliations:** 1 Institut für Physik, Johannes Gutenberg-Universität Mainz, Staudingerweg 7-9, 55099 Mainz, Germany; 2 College of Integrated Circuits and Micro-Nano Electronics, Fudan University, Handan Road 220, Shanghai, 200433 China; 3 Physics of Complex Fluids, MESA+ Institute, University of Twente, PO Box 217, 7500AE Enschede, The Netherlands

## Abstract

**Abstract:**

We investigate the kinetics of wetting ridge growth and droplet cloaking on lubricant-infused polymer brushes using a combination of experiments, molecular dynamics simulations, and theoretical modeling. We focus on three representative systems: DMSO-water on hexadecane-swollen PLMA (D-H), water on hexadecane-swollen PLMA (W-H), and water on PDMS (W-S). The dynamics are governed by the interplay between interfacial thermodynamics, brush elasticity, and transport of lubricant within the brush. Ridge growth is accompanied by the formation of depletion zones both beneath and outside the drop. This leads to a progressive slowdown governed by the need to transport lubricant through the brush. At sufficiently high swelling, we observe local separation of oil from the brush within the ridge, providing an additional mechanism for lubricant depletion. To rationalize these observations, we develop a continuum diffusion model based on the free energy of the brush and its coupling to the contact line. The model quantitatively captures the growth of the wetting ridge at intermediate and late times, demonstrating that the kinetics are largely controlled by diffusive transport within the brush.

**Graphical abstract:**

**Figure:**
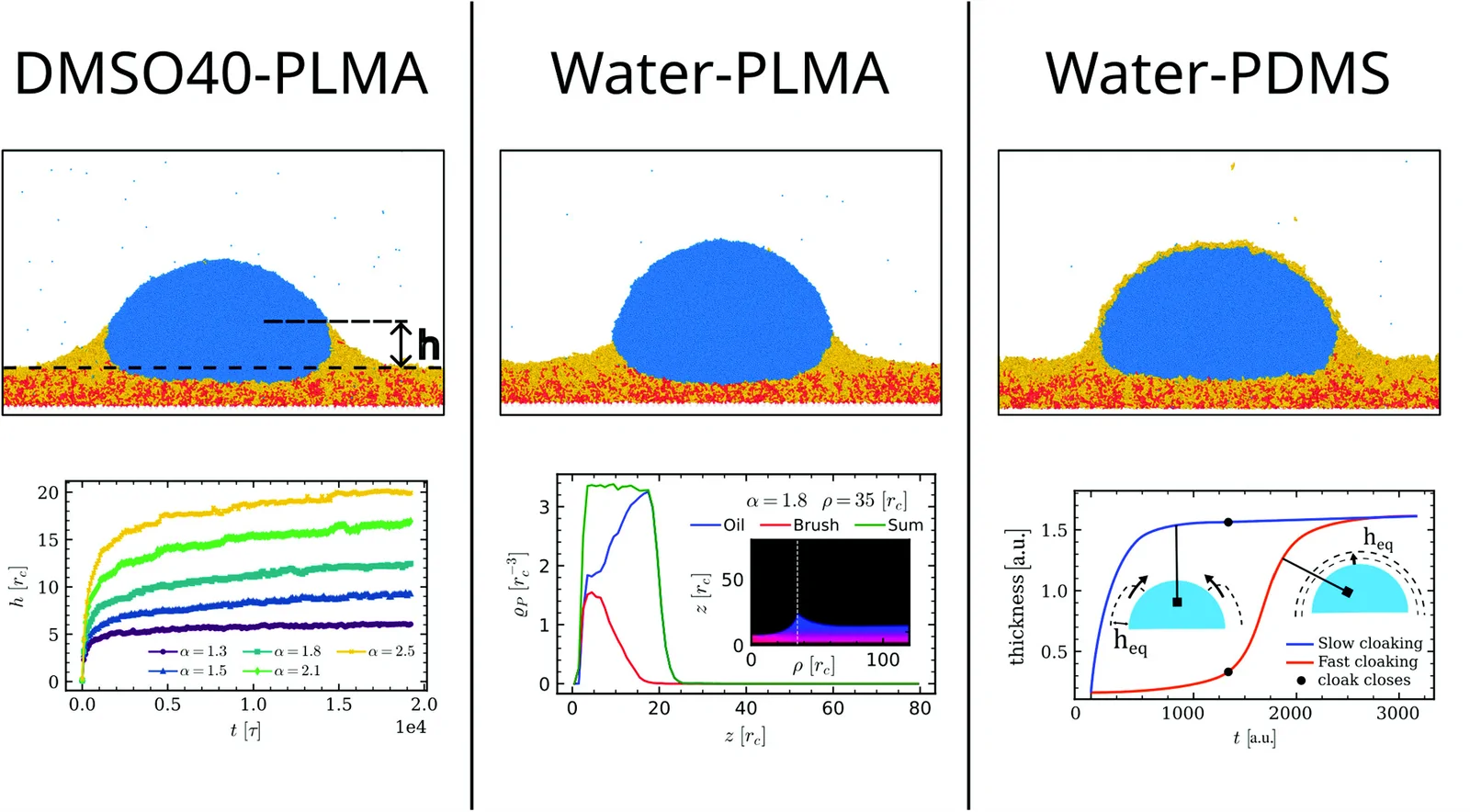

**Supplementary Information:**

The online version contains supplementary material available at 10.1140/epje/s10189-026-00607-6.

## Introduction

Polymer brushes are widely used to modify solid surfaces, with applications ranging from adhesion reduction and lubrication enhancement [1–6] to diagnostics [7, 8] and biosensing [9], among others. In many of these contexts, the interaction of polymer brushes with liquids, particularly liquid drops, is essential. Polymer brushes exhibit several advantageous properties. They can adapt to the presence of drops [10, 11] or respond to external stimuli [12, 13] and change their wetting properties accordingly. In addition, drops on polymer brushes can have low contact angle hysteresis, while the coatings themselves are highly durable and retain lubricants very well [3, 14]. The last property is central for applications such as ball bearings and self-cleaning surfaces, where the brush acts as a reservoir for oil lubricants that reduce friction [3, 15, 16]. The desirable properties of polymer brushes interacting with liquid drops arise from the interplay between brush elasticity, interfacial properties, and the presence of liquid lubricant within the brush.

Much effort has been devoted to understanding the interaction of liquid drops with lubricant-infused surfaces [17–23] and elastomers [24–26]. More recently, particular attention has been given to lubricant-infused polymer brushes [3, 27, 28]. A key question in such systems is whether the lubricant is extracted from the brush in the presence of a drop. Interfacial forces at the three-phase contact line can drive oil separation, leading to the formation of a two-phase wetting ridge [26] or even complete cloaking of the drop [27, 29, 30]. These processes contribute to lubricant depletion on the substrate and, consequently, to the degradation of surface functionality [3, 18, 19]. Moreover, the presence of a liquid phase in the wetting ridge can influence drop dynamics by modifying dissipation mechanisms [31].

In this work, we investigate the kinetics of lubricant redistribution during the growth of wetting ridges and the formation of cloaking layers. We adopt a combined experimental, theoretical, and simulation-based approach. Experimentally, we study aqueous dimethyl sulfoxide (DMSO) drops deposited on poly-lauryl methacrylate (PLMA) brushes swollen with hexadecane and track the time evolution of the wetting ridge. These investigations are complemented by molecular dynamics simulations of an analogous system. We further use simulations to investigate oil separation in water-PLMA brush systems and to quantify the progression of the nanoscale cloaking layer in a water-PDMS system. Finally, motivated by the observed redistribution of lubricant, we develop a theoretical framework based on a diffusion equation derived from the free energy of the brush and the contact line.

## Model and methods

We consider systems in which a liquid drop is deposited on a lubricated polymer brush. The primary experimental systems consist of PLMA brushes swollen with hexadecane and PDMS brushes swollen with silicone oil. In both cases, the monomers of lubricant and grafted chains can be regarded as chemically identical, and we treat them as such in our simulations. However, in both experiments and simulation, the number of monomers per oil chain $N_o$ is much smaller than that of grafted chains $N_B$. In particular, we have $N_B > N_o^2$ for all of our systems, which implies that the lubricant acts as an athermal solvent for the grafted chains [32]. While water is the primary liquid of interest, aqueous DMSO solutions (DMSO40 corresponding to 40 wt % concentration by weight) on PLMA are also considered to probe lower surface tension conditions. Table 1 shows the experimental values for the relevant interfacial tensions (values for PDMS extracted from Ref. 27).

**Table 1:** Experimental interfacial tension values of the different interfaces that appear in our systems of interest

(mN/m)	Air	PDMS	Hexadecane
Air		21	28.76±0.18
Water	72.6±0.8	40	50.4±0.4
DMSO40	62.3±0.9		31.4±0.6

Simulation parameters are chosen to reproduce the ratios of interfacial tensions in the experimental systems, ensuring realistic contact angles and spreading behavior.

### Simulations

We employ a coarse-grained model system comprising a polymer brush, free oil chains, and a droplet of liquid particles coexisting with its vapor. Brush polymers and oil molecules are modeled as chains of identical beads, denoted *p*, connected by springs with the spring potential $U_{\text {bond}}={\frac{1}{2}}k(r_{ij}-r_0)^2$. Liquid molecules are modeled as single isolated beads (type *l*). To model non-bonded interactions and the coupling to a heat bath at a given temperature *T*, we use the Many-body Dissipative Particle Dynamics (MDPD) coarse-grained model and thermostat. The DPD thermostat has the advantage that the dissipative and random forces are pairwise interactions and momentum conserving allowing for hydrodynamic phenomena [33–35], while the multi-body force element allows for modeling the coexistence of two phases [36–38]. The forces take the following form:

1 $$\begin{aligned}&\textbf{F}_{ij}=\textbf{F}_{ij}^C+\textbf{F}_{ij}^D+\textbf{F}_{ij}^R \end{aligned}$$

2 $$\begin{aligned}&\textbf{F}_{ij}^C=\left( A_{ij}w^C(r_{ij})+B(\bar{\rho }_i+\bar{\rho }_j)\tilde{w}^C(r_{ij})\right) \hat{\textbf{r}}_{ij} { + \textbf{F}_{ij}^{\text {bond}}} \end{aligned}$$

3 $$\begin{aligned}&\textbf{F}_{ij}^D=-\zeta w^C(r_{ij})^2(\hat{\textbf{r}}_{ij}.\textbf{v}_{ij})\hat{\textbf{r}}_{ij}\end{aligned}$$

4 $$\begin{aligned}&\textbf{F}_{ij}^R=\sqrt{2\zeta k_B T}w^C(r_{ij})\theta _{ij}\hat{\textbf{r}}_{ij}\end{aligned}$$

5 $$\begin{aligned}&w^C(r_{ij})={\left\{ \begin{array}{ll} \bigg (1-\frac{r_{ij}}{r_c}\bigg )~~& r_{ij}\le r_c\\ 0~~& r_{ij}>r_c \end{array}\right. } \end{aligned}$$

6 $$\begin{aligned}&\tilde{w}^C(r_{ij})={\left\{ \begin{array}{ll} \bigg (1-\frac{r_{ij}}{r_d}\bigg )~~& r_{ij}\le r_d\\ 0~~& r_{ij}>r_d \end{array}\right. }\end{aligned}$$

7 $$\begin{aligned}&\bar{\rho }_i=\sum _{j\ne i}\frac{15}{2\pi r_d^3}\tilde{w}^C(r_{ij})^2 \end{aligned}$$

In the above equations, $\textbf{F}_{ij}^C$ is the conservative force contribution where $A_{ij}<0$ is the strength of the non-bonded attractive part, B>0 is the strength of the density-dependent repulsion, and the spring forces $\textbf{F}_{ij}^{\text {bond}} =- k (r_{ij}-r_0) \hat{\textbf{r}}_{ij}$ act only between neighbor monomers on a chain. *B* must have the same value for all pairs of particles for the forces to be conservative as shown by the no-go theorem of MDPD [39]. We also have $\textbf{r}_{ij}=\textbf{r}_i-\textbf{r}_j$, and $\hat{\textbf{r}}_{ij}=\textbf{r}_{ij}/r_{ij}$. $\textbf{F}_{ij}^D$ and $\textbf{F}_{ij}^R$ are the dissipative and random force contributions, respectively, where ζ is the drag coefficient, $\textbf{v}_{ij}=\textbf{v}_i-\textbf{v}_j$, $k_B$ and *T* are Boltzmann’s constant and the temperature, respectively, and $\theta _{ij}$ is an uncorrelated Gaussian distributed random variable with zero mean and unit variance. $w^C$ and $\tilde{w}^C$ are weight functions, $\tilde{\rho }_i$ is a weighted density, and finally $r_c$ and $r_d$ are cutoff radii which set the range of the forces. The reason for introducing two cutoff radii is that the range of the density-dependent repulsion must be smaller than that of the attraction, $r_d<r_c$, (with $A_{ij} < 0$ and B>0) in order to obtain liquid–vapor coexistence [38].

The polymer brush consists of end-grafted chains of length $N_B$. The chains are grafted to a purely repulsive surface modeled using the Weeks-Chandler-Anderson (WCA) potential [40]. The oil is modeled as free chains of length $N_o$. As already mentioned, monomers in the free oil chains and the grafted brush chains are all taken to be of the same species (*p*) and therefore have the same interaction parameters among each other and with the liquid particles.

The simulations are performed at constant number of particles, volume, and temperature, i.e., in the canonical NVT ensemble, using periodic boundary conditions in all directions. The unit of energy is set by $$k_BT=1$$, the unit of length by the cutoff distance of the DPD attraction $$r_c=1$$, and the mass unit by m=1 for all species. The unit of time can then be defined as $$\tau =\sqrt{\frac{m r_c^2}{k_BT}}$$. In the following, all quantities are given in these units. The model parameters were chosen to be the same as in our previous work [27], with the DPD parameters $$r_d=0.8;~\zeta =4.5;~ B=40$$ (see below for a discussion of the choices of the parameters $$A_{ij}$$), and bond potential parameters $$ k=20, r_0=1$$. From the balance of the bonded and non-bonded forces, the resulting average bond length is a≈1.09 with a standard deviation of $$\sigma _a = 0.22$$ based on an average over $$10^6$$ bonds. The same numbers are obtained for ideal chains with non-bonded forces turned off, indicating that non-bonded interactions have no influence on the bond length distribution. For the WCA potential of the wall where chains are grafted we choose $$\sigma _{WCA}=1,\epsilon =1$$. With this choice of parameters and the thickness of the brush, the wall to which the chains are attached does not directly influence the wetting behavior of the liquid. Instead, the wetting properties are purely determined by the interactions of the droplet with the grafted and lubricant chains. All simulations are performed in the absence of any gravitational forces. The time step of the simulation was chosen $$~\Delta t=10^{-3}$$.

**Table 2:** Values for the parameters $A_{ij}$ of the attractive DPD force for the different systems we simulate

	$A_{pp}$	$A_{ll}$	$A_{pl}$
W-S	−28	−50	−21
W-H	−32.5	−53	−20
D-H	−32.5	−50	−21.5

A subscript ‘*p*’ corresponds to polymer, while ‘*l*’ corresponds to liquid, and two letters correspond to the interaction between the two species

**Fig. 1:**
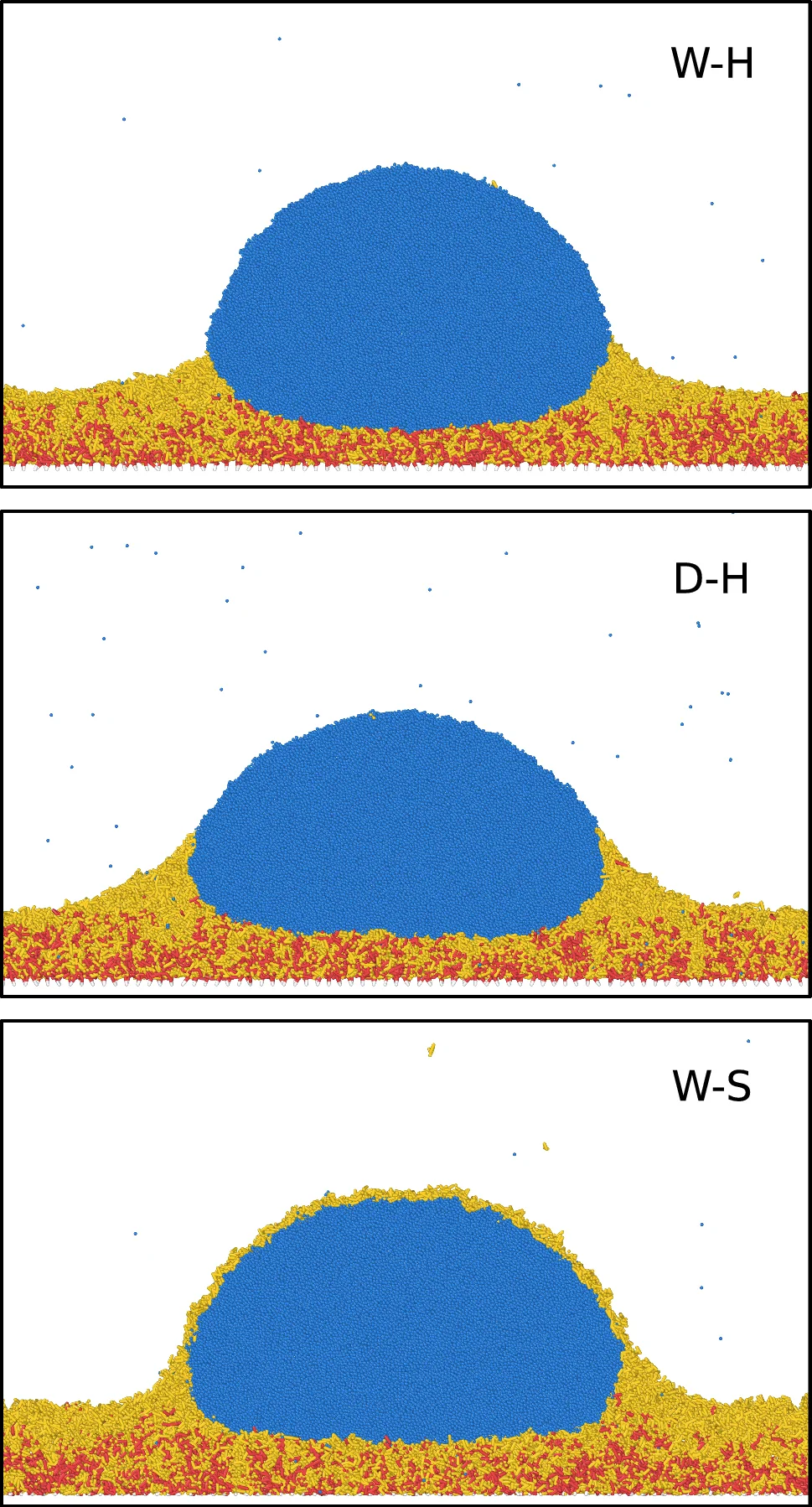
Snapshots from simulations of the systems analogous to water on PLMA, DMSO40 on PLMA, and water on PDMS. Yellow chains are oil, red are grafted chains, and blue particles constitute the liquid. The snapshots for the W-H and D-H systems do not correspond to the equilibrium configurations

To produce systems mimicking the experimental systems, we chose the interaction parameters such that the surface tensions between the different phases in simulation reproduce the relevant contact angles, i.e., contact angle of the liquid drop on a bulk of the brush material under ambient conditions. This sets our choices of the DPD parameters for the polymer–polymer cohesion $A_{pp}$, the liquid–liquid cohesion $A_{ll}$, and the polymer–liquid adhesion $A_{pl}$. Since we have three different combinations of drop and brush, we introduce a shorthand notation to refer to each combination. We use W-S (water-silicone) to refer to the water on PDMS and silicone oil, W-H (water-hexadecane) to refer to water on PLMA and hexadecane, and D-H (DMSO40-hexadecane) to refer to DMSO40 on PLMA and hexadecane. Table 2 shows the final values of the $A_{ij}$ parameters for the different systems. Our choice for the W-S system is motivated by our choice in previous work [15, 27]. As for the W-H system, to match the contact angles, we increased both the liquid–liquid cohesion strength $A_{ll}$ and the polymer–polymer cohesion $A_{pp}$, although the drop is still supposed to represent water. The other option was to reduce $A_{pp}$ while keeping $A_{ll}$ constant; however, this lead to a much larger vapor pressure for the oil and destabilized the system. Therefore, we opted to increase $A_{pp}$ and used the same value for the W-H and D-H systems, while tuning the values of $A_{ll}$ and $A_{pl}$ to match the contact angles. Each choice for the set of attraction strengths determines the densities, interfacial tensions, and viscosities of the different species and interfaces. Table S.1 summarizes the resulting values of the physical quantities for each system of interest, and Fig. 1 shows some representative snapshots. The surface tension and viscosity are calculated as described in Refs. 27 and 15 (SI), respectively.

The systems for simulation are prepared as described in section S.5.1. The simulations are conducted using the HOOMD-Blue simulation package [41, 42] version 4.5.0. All snapshot visualizations are made with the OVITO visualization package [43]. Details of certain analysis methods are described in section S.5.

### Experiments

Experiments were carried out using polymer brushes grown by surface-initiated activators regenerated by electron transfer radical polymerization (SI-ARGET-ATRP) on microscope cover slips following the method described in refs. [44, 45]. The dry thickness of the brushes is $160\pm 5 {\textrm{nm}}$. Brushes were impregnated with a fluorescent dye (nile red) and pre-swollen by exposing them to hexadecane vapor for up to 7 min, leading to a variable degree of swelling with swelling ratios in the range α=1...4. The actual experiments were initiated by depositing a millimeter-sized drop of a 40-60% weight/weight DMSO-water onto the pre-swollen brush layer. Drop deposition was followed by a quick spreading process within $\sim 1\textrm{s}$ to a steady drop shape with a macroscopic contact angle of $\approx 90^{\circ }$. More details will be reported elsewhere [46].

### Continuum model

To describe the dynamics of oil transport, we construct a continuum model based on the free energy of the brush and its coupling to the contact line. The model is formulated in terms of the local volume fraction of oil, $\varphi (\textbf{r})$. We begin by defining the free energy of the brush as a functional of $\varphi (\textbf{r})$,

8 $$\begin{aligned} \frac{\mathcal {F}_B[\varphi (\textbf{r})]}{k_B T} = \int \text {d}V \, \frac{f_B(\varphi (\textbf{r}))}{{k_B T}}, \end{aligned}$$

where $f_B(\varphi (\textbf{r}))$ is a free energy density. Assuming azimuthal symmetry and fast vertical equilibration compared to lateral transport, we take the concentration to depend only on the radial coordinate ρ. Under this assumption, the free energy density is written as

9 $$\begin{aligned} \frac{f_B(\varphi (\rho ))}{{k_B T}} = \frac{\varphi }{N_o {a^3}} \ln {\varphi } + \frac{\chi (\varphi )}{N_o {a^3}} \varphi \left( 1-\varphi \right) + \frac{k}{2} \frac{\sigma ^2 {a}}{1-\varphi }, \end{aligned}$$

where $N_o$ is the number of monomers per oil chain, *a* is the size of a monomer, $\chi (\varphi )$ is a dimensionless interaction function, *k* is the dimensionless elastic constant of the brush, and σ is the grafting density of the brush with unit $[L]^{-2}$. The first term in Eq.  9 represents the translational entropy of the oil molecules, the second term accounts for interactions between oil and grafted chains through a concentration-dependent interaction parameter $\chi (\varphi )$ [47, 48], and the third term captures the elastic penalty associated with stretching the grafted polymers. The function $\chi (\varphi )$ is not known *a priori* but can be determined following an approach similar to that of Schubotz et al. [48]; details of the calculation for our simulated brushes can be found in Sec. S.3. Most importantly, when the brush is saturated, we expect the lubricant to act as an athermal solvent since we have $N_B > N_o^2$ [32]. Therefore, we construct our interaction function to vanish when the brush is saturated, so that we recover the free energy of a brush with an athermal solvent, namely χ=0.

In addition to the free energy of the brush, we include the contribution of the three-phase contact line through a free energy line density $f_{\text {cl}}(\lambda )$ where λ is the line density of oil at the three-phase contact line. The corresponding contribution to the total free energy $\mathcal {F}_{\text {cl}}$ is

10 $$\begin{aligned} \frac{\mathcal {F}_{\textrm{cl}} [\lambda ]}{{k_B T}} = \oint \text {d}l \, \frac{f_{\textrm{cl}}(\lambda )}{{k_B T}}, \end{aligned}$$

where the integral is evaluated along the three-phase contact line. We assume a quadratic form for the line free energy density

11 $$\begin{aligned} \frac{f_{\textrm{cl}}(\lambda )}{{k_B T}} = \frac{\kappa }{2} \left( \lambda - \lambda _0 \right) ^2, \end{aligned}$$

where κ is an effective stiffness, and $\lambda _0$ is the saturation line density. In the following, we set $k_B T=1$ and a=1. The dynamics in the system is driven by the difference in chemical potential between the contact line and the brush. At the contact line, we assume that the rate of change of the line density λ is proportional to the chemical potential difference and to the local oil availability. This leads to the evolution equation

12 $$\begin{aligned} \frac{\partial \lambda }{\partial t} = -\mathcal {B} \Phi (\rho =R_{\textrm{cl}}) \left[ f_{\textrm{cl}}'(\lambda ) - \mu _B(t) \right] , \end{aligned}$$

where $\mathcal {B}$ is a filling rate, $\Phi (\rho ) = \int \varphi (\rho ) \, \text {d}z$, and $R_{\textrm{cl}}$ is the radial position of the contact line. To simplify the picture, we further assume that the chemical potential in the brush is always close to its equilibrium value $\mu _B^{\textrm{eq}}$. This allows to rewrite the evolution equation for λ, Eq. 12, as

13 $$\begin{aligned} \frac{\partial \lambda }{\partial t} = -\mathcal {B} \Phi (R_{\textrm{cl}}) \kappa \left( \lambda - \lambda _e \right) \end{aligned}$$

with the equilibrium line density

14 $$\begin{aligned} \lambda _e = \lambda _0 + \frac{\mu _B^{\textrm{eq}}}{\kappa }, \end{aligned}$$

which depends on the equilibrium oil fraction $\varphi ^{\textrm{eq}}$ in the brush.

To describe transport within the brush, we consider radial currents on either side of the contact line at $\rho = R_{\textrm{cl}}$. The radial flux is expressed as

15 $$\begin{aligned} j_\rho = -M \varphi \nabla _\rho \frac{\delta \mathcal {F}_B \left[ \varphi \right] }{\delta \varphi }, \end{aligned}$$

where *M* is the mobility of oil in the brush, $\nabla _\rho$ is the radial component of the gradient operator in cylindrical coordinates, and $\delta / \delta \varphi$ denotes the variational derivative with respect to the concentration profile $\varphi (\rho ,z)$. The evolution of the concentration field then follows from the continuity equation

16 $$\begin{aligned} \frac{\partial \varphi }{\partial t} = - \nabla _\rho j_\rho . \end{aligned}$$

The full equations are presented in full detail in SI.

To solve the equations, appropriate boundary conditions are imposed. At the origin (ρ=0), symmetry requires

17 $$\begin{aligned} j_\rho (0,t) = 0. \end{aligned}$$

At the outer boundary layer, the brush is assumed to be in contact with a reservoir that fixes the oil fraction,

18 $$\begin{aligned} \varphi (L,t) = \varphi _B. \end{aligned}$$

At the contact line ($\rho = R_{\textrm{cl}}$), conservation of mass requires the rate of change of the line density to equal the net flux from both sides

19 $$\begin{aligned} \frac{\partial \lambda }{\partial t} = - H(\varphi (R_{\textrm{cl}})) \left( j_\rho ^{>}(R_{\textrm{cl}}) - j_\rho ^{<}(R_{\textrm{cl}}) \right) \end{aligned}$$

where $H(\varphi (R_{\textrm{cl}}))$ is the brush height at the contact line and $j_\rho ^{<}(R_{\textrm{cl}})$, $j_\rho ^{>}(R_{\textrm{cl}})$ refers to the fluxes at the inner and outer side of the contact line, respectively.

For numerical implementation, the radial domain is discretized into bins of width Δr, with positions $r_i$. Spatial derivatives are approximated using finite differences,

20 $$\begin{aligned}&\frac{\partial g_i}{\partial r} = \frac{1}{2 \Delta r} ( g_{i+1} - g_{i-1} )\end{aligned}$$

21 $$\begin{aligned}&\frac{\partial ^2 g_i}{\partial r^2} = \frac{1}{\Delta r^2} ( g_{i+1} + g_{i-1} - 2 g_{i} ) \end{aligned}$$

and time integration is performed using a forward Euler scheme. The boundary conditions are implemented numerically by enforcing

22 $$\begin{aligned} \varphi _0(t) = \varphi _1(t), \qquad \varphi _L(t) = \varphi (L,t) = \varphi _B. \end{aligned}$$

To impose the conservation of mass at the contact line, we discretize Eq. 19 using forward and backward expressions for finite derivatives. This results in an algebraic equation that we can solve for the fraction of oil at the contact line $\varphi (R_{\textrm{cl}})$. The full form of the equation we need to solve is given in Eq. S.56. The model contains several parameters that can be extracted from simulations, including the elastic constant *k*, the interaction function $\chi (\varphi )$, and the equilibrium line density $\lambda _e$. The procedures to determine these parameters are described in Sec. S.5.6. The mobility *M* and the kinetic coefficient $\mathcal {B}$ are treated as fitting parameters and adjusted to match the simulation results.

**Fig. 2:**
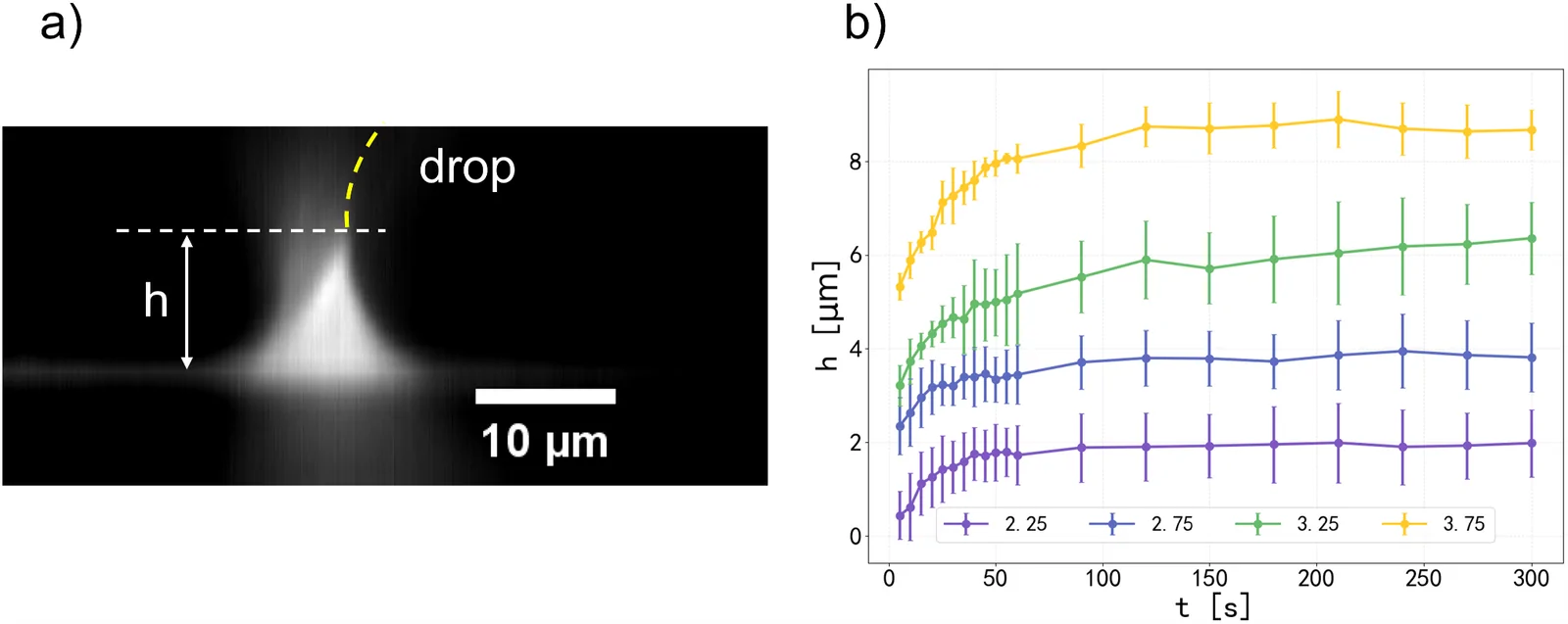
**a** Confocal fluorescence microscopy image of a wetting ridge for a DMSO40 drop on a PLMA brush swollen with hexadecane after 300 s; swelling ratio: α=3.25. *h* indicates of apex of the wedge above the brush. **b** Evolution of the height of the ridge with time for brushes with different swelling ratios (see legend). t=0 indicates the time of the first snapshot after drop deposition and spreading, which involves some wedge growth for higher degrees of initial swelling

## Results and discussion

### Kinetics of ridge and cloak development

We first examine the kinetics wetting ridge growth and cloaking. Ridge growth is quantified by the evolution of the height of the ridge above the unperturbed brush. For water on hexadecane (W-H), we also analyze oil separation within the ridge. For water-PDMS systems (W-S), which exhibit clear signs of cloaking, we focus on the propagation of the cloak front and the thickening of the cloaking layer.

#### DMSO40 on PLMA

We begin by discussing the results for DMSO40 on PLMA brushes, swollen with hexadecane. The spreading parameter of the oil on the drop in this case is positive $S_{p/l} = 2.1 \pm 1.1 \, \mathrm {mN/m}$. Therefore, a cloaking layer could form on the drop. Figure 2 shows the experimental results for a growing oil wedge for this system. Figure 2a shows a typical confocal microscopy image of the oil forming a wetting ridge around the DMSO40 drop for a brush with a swelling ratio of α=3.25. The ridge appears as a triangular fluorescent wedge with the drop on its right side; the height is measured as the distance between the surface of the brush and the apex of the wedge. Figure 2b shows the evolution of the height of the ridge with time, where the error bars are standard errors over 4 repetitions of the experiment. The height grows gradually with time and seems to saturate at a maximum height that increases with the swelling ratio. Despite the positive spreading parameter of hexadecane on the DMSO40 solution, we do not see signs of cloaking. This could either indicate that the saturation level of the brush is still below the cloaking transition [27], or that the microscope cannot resolve the presumably very thin cloaking layer. However, the experiments show signs of separation of the oil from the brush, as the maximum height of the wedge exceeds the maximum height of the fully swollen brush by more than a factor of 10 for the most swollen brushes. Given the positive spreading parameter of the oil on the DMSO40 solution this is not surprising; it is nonetheless an interesting observation since the separation may play a role in the depletion of oil from the system.

**Fig. 3:**
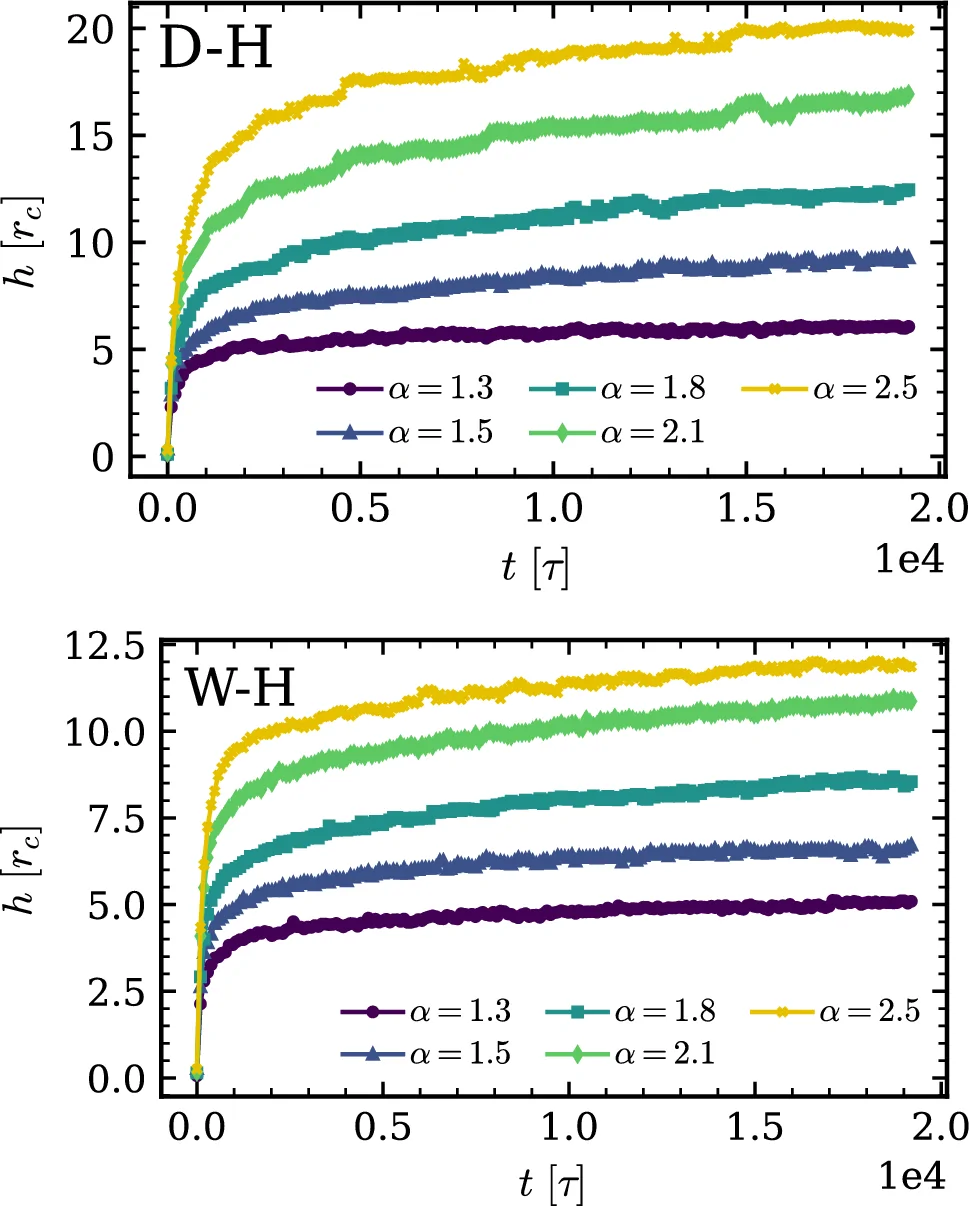
Height above the brush reached by the apex of the ridge versus time for the D-H and W-H systems at different swelling ratios

To gain more insight on the molecular scales, and investigate the possible reason for the absence of a cloak, we run molecular dynamics (MD) simulations of a system mimicking the DMSO40 drop on a PLMA brush swollen with hexadecane (D-H). As described above, the interaction parameters are chosen to match the experimental contact angle and the sign of the spreading parameter. It should be noted, however, that the swelling ratios accessible in simulation are lower than those in experiments, owing to the significantly shorter chain lengths employed in the coarse-grained model. The maximum swelling ratio for our PLMA-like brushes is $\alpha ^*\approx 2.5$. The ridge height in simulations is determined analogously to the experimental procedure (see Sec. S.5.3). The temporal evolution of the ridge height for the D–H system is shown in Fig. 3a. The results indicate that the system has not reached equilibrium within the accessible simulation times, except possibly at the lowest swelling ratio. Notably, even for an oversaturated brush with $\alpha = \alpha ^*$, no cloaking of the DMSO40 drop is observed within the simulation window. This suggests that cloaking is kinetically hindered and that the system has not evolved for a sufficiently long time to reach its equilibrium configuration. The combined effect of the brush chains being longer than the lubricant chains and the fact that they are grafted can lead to an effectively more viscous environment in the brush. This in turn can result in slower diffusion of the lubricant in the brush when compared to a medium of pure lubricant. In fact, experimental investigations of the diffusion of hexadecane in PLMA brushes show that it is much slower compared to pure oil [46].

To test this hypothesis, we perform additional simulations in which oil droplets are brought into contact with liquid droplets in the absence of grafted chains, thereby removing the constraints imposed by the brush. The final configurations after a simulation time of $6.4 \times 10^3,\tau$ (Fig. S.8) clearly show that, for the D–H system with a positive spreading parameter ($S_{p/l} > 0$), the oil fully engulfs the liquid, resulting in a core–shell morphology. This observation confirms that cloaking is thermodynamically favorable in this system. We therefore expect that, in the presence of the brush, a fully cloaked state would eventually be reached at sufficiently large times and swelling ratios. However, accessing these regimes remains computationally prohibitive within the present study.

#### Water on PLMA

**Fig. 4:**
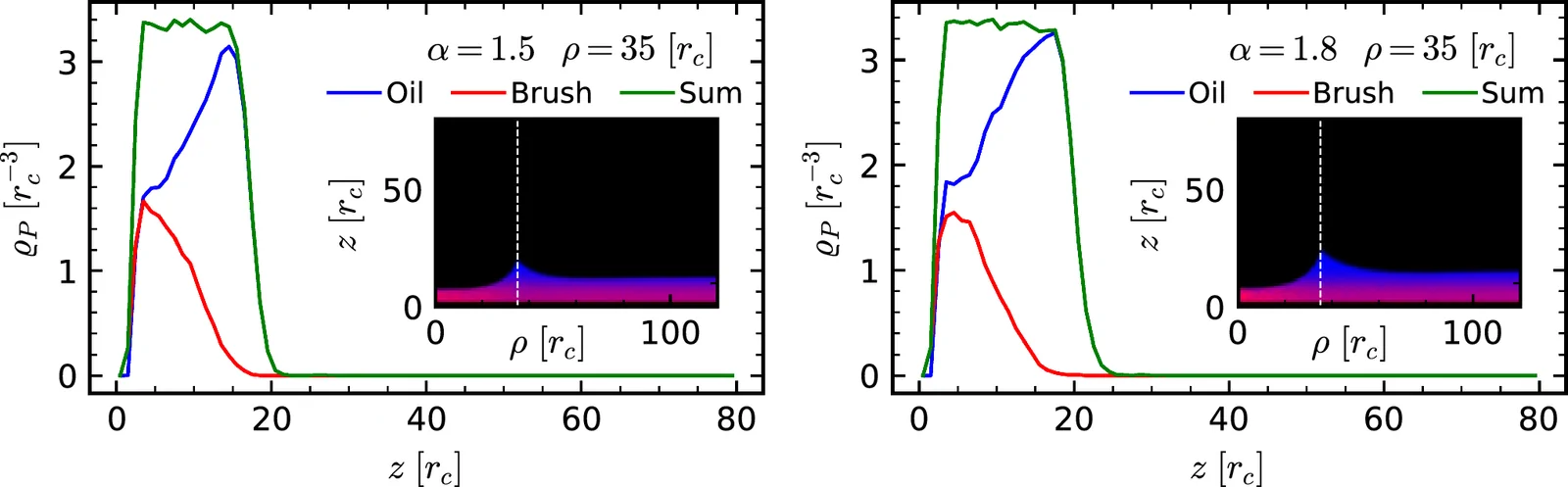
Vertical density profile of polymers in the W-H system in the area of the wetting ridge at radial position $\rho =35 \, [r_c]$ for swelling ratios α=1.5 and α=1.8. Insets show the density map $\varrho (\rho ,z)$ with the grafted chains in red and the oil in blue. Magenta indicates a mixture of the two. Although the insets suggest that far from the droplet, the brush is covered by a layer of oil, the profiles show that is not the case—the change of color merely reflects the accumulation of oil in the polymer/vapor interface without the formation of a bulk oil layer (see Figure S.7). The dashed lines indicate the position where the density profile is shown. For the higher swelling ratio we clearly see that the fluid separates from the brush as indicated by a density of oil equal to the bulk density of polymers

Since water is a prevalent liquid in many applications, it is of particular interest to investigate the interaction of water drops with lubricated polymer brushes. Experimentally, however, this system is difficult to probe using top-view microscopy, as the large contact angle of water on PLMA (exceeding 100∘) obscures direct observation of the wetting ridge. We therefore restrict our analysis of this system to MD simulations.

Figure 3b shows the time evolution of the ridge height for systems corresponding to water on a PLMA brush swollen with hexadecane (W–H) at different swelling ratios. As in the D–H system, the ridge height increases with time and does not fully saturate within the accessible simulation window, except at the lowest swelling ratio. However, at comparable swelling, the ridge height in the W–H system is consistently smaller than in the D–H case. Since the spreading parameter of oil on water in the W-H system is negative ($S=-0.43 \pm 0.06 \, [k_BT/r_c^2]$), cloaking is not expected. In this case, we expect the oil and liquid to adopt a Neumann configuration for the angles (see section S.2). Based on our values for the surface tension in simulation, we expect a triplet of angles $(161.42^\circ ,143.01^\circ ,55.57^\circ )$. Since the simulations with the brush did not reach equilibrium, we calculate the contact angles between oil and liquid from contacting droplet simulations (see Figure S.8). The values of the angles calculated from those simulations are $(164.06^\circ ,137.82^\circ ,58.12^\circ )$. The values from the simulations agree reasonably with the values obtained from the Neumann balance. Despite the absence of cloaking, inspection of simulation snapshots (see Fig. S.8) suggests that the oil can partially separate from the brush within the wetting ridge. Since such separation may enhance lubricant depletion, it is important to characterize this phenomenon in more detail.

Oil separation has previously been reported and quantified for water drops on PDMS elastomers swollen with silicone oil [26, 49], where the spreading parameter is positive and cloaking can occur. In that context, separation is a necessary precursor to cloaking, although it has also been observed in cases where no visible cloak forms [26, 49]. In contrast, for systems with a negative spreading parameter, such as hexadecane on water, the occurrence of oil separation is less straightforward and requires careful analysis.

To address this, we examine the final configurations of the W-H system at different swelling ratios. The maximum swelling ratio for the PDMS-like brush is $\alpha ^*\approx 2.3$ Figure 4 shows the vertical density profiles $\varrho (\rho _{\textrm{ridge}},z)$ of grafted chains, oil, and their sum at the position of the wetting ridge, (radial coordinate $\rho _{\textrm{ridge}} = 35 \, [r_c]$, see white dashed line in the inset), for swelling ratios α=1.5 and α=1.8. The insets display the full density map of polymers $\varrho _P(\rho ,z)$, where grafted chains are shown in red and oil in blue; mixed regions appear in magenta. At the lower swelling ratio (α=1.5), the oil remains well mixed with the brush throughout the ridge region, as indicated by the oil density remaining below the total polymer density. In contrast, at the higher swelling ratio (α=1.8), the oil density near the top of the ridge approaches the total density, indicating the onset of phase separation. Additional data for other swelling ratios (see Fig. S.6) confirm this trend. Moreover, density profiles taken far from the drop (ρ=80, S.7) show that, for undersaturated brushes, the oil remains fully incorporated within the brush. This observation also informs the proper reading of the colored density maps in the insets of Figs. 4, S.6, and S.7. One may be tempted to see a blue color and conclude that the oil separated from the brush. However, this is an optical effect, and the true nature of the separation can only be concluded from the density profiles.

These results demonstrate that the presence of a water drop can induce local separation of oil from the brush within the wetting ridge, even in the absence of cloaking, which in turn affects the rate of oil depletion in applications. This separation only occurs after the brush is sufficiently swollen.

#### Water on PDMS

When the polymer brush consists of PDMS swollen with chemically identical silicone oil, the spreading parameter of the oil on a water drop is significantly positive ($S_{p/l} \approx 12 \mathrm {mN/m}$). Under these conditions, cloaking of the drop sets in once the brush is sufficiently swollen  [27]. We therefore focus on quantifying the kinetics of cloak formation. To this end, we first track the evolution of the cloaking layer by following the position of the cloak front, which gradually advances toward the apex of the droplet.

To find the position of the cloak front, we calculate the azimuthally symmetric local density of oil $\varrho _o(r,\theta )$ in spherical coordinates as described in section S.5.2 (see Fig. S.2b). From the density map, we find the equal density contours $\varrho _o(r,\theta ) = \rho _o/2$. Afterward, we calculate the angular position reached by the cloak as the smallest angular value on the contour. The arc length covered by the cloak can then be calculated as

23 $$\begin{aligned} s(t)= (\theta _{\textrm{app}}(t)-\theta _{\textrm{front}}(t)) \times R(t), \end{aligned}$$

where $\theta _{\textrm{app}}$ is the apparent contact angle of the drop, $\theta _{\textrm{front}}$ is the polar angle reached by the cloak front, and $R_{\textrm{D}}$ is the radius of curvature of the drop.

**Fig. 5:**
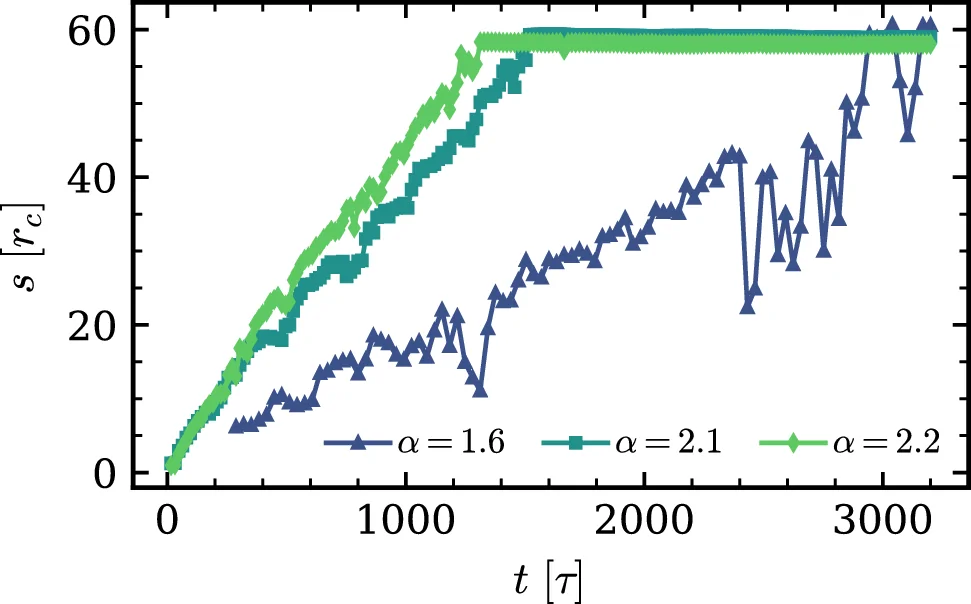
Cloak front position versus time for three different swelling ratios α, all beyond the cloaking transition. As expected the higher the swelling ratio, the faster the cloaking progresses

The temporal evolution of the cloak front is shown in Fig. 5 for several swelling ratios above the cloaking threshold. In all cases, the cloak front advances approximately linearly in time, indicating a nearly constant propagation speed. Moreover, the speed increases with increasing swelling ratio.

**Fig. 6:**
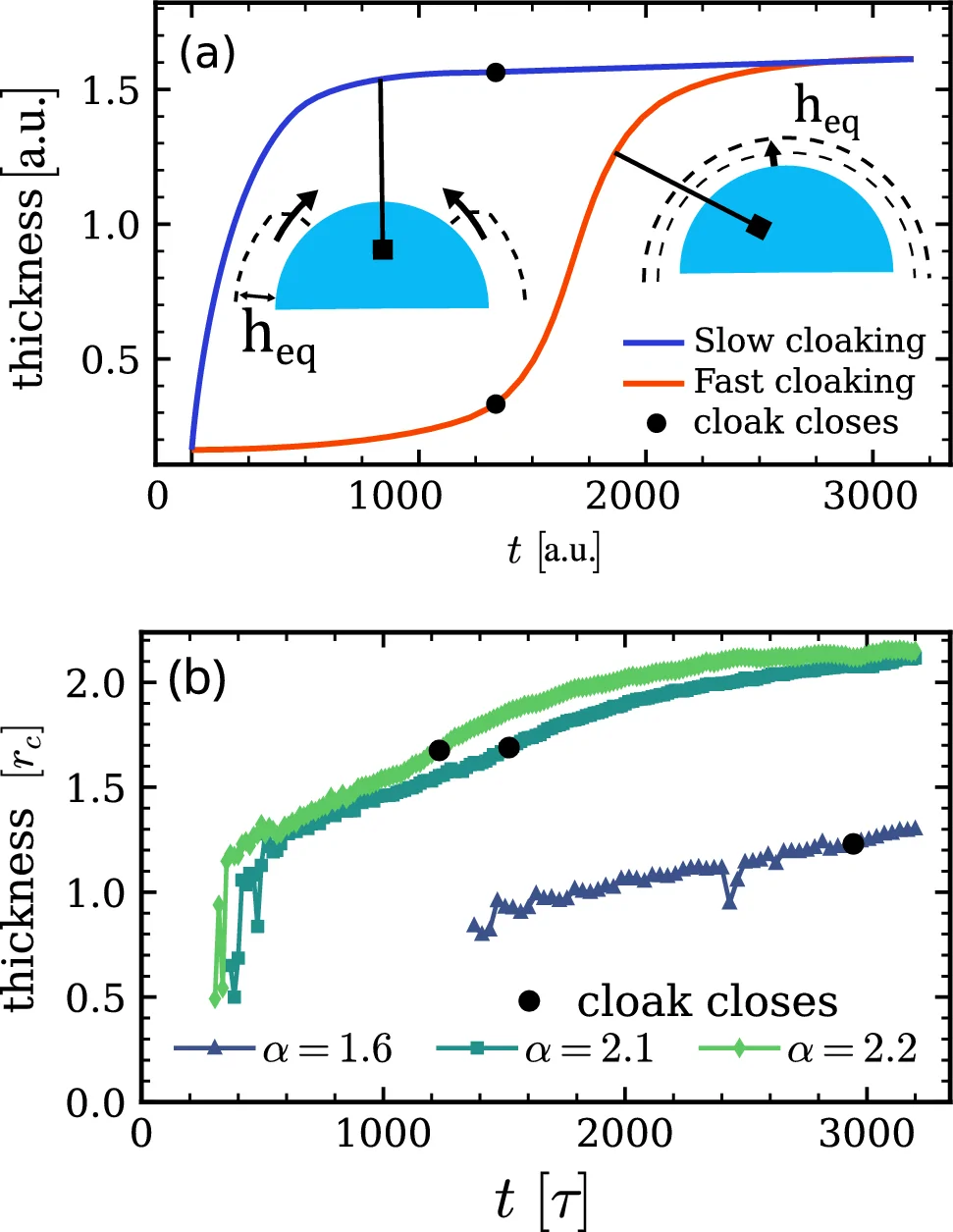
**a** Qualitative representation of the two extreme thickening regimes of the cloak. **b** Thickness of the cloak versus time for the three swelling ratios we studied. In both figures the black dots refer to the time and thickness when the cloak reaches the top of the droplet

Beyond the position of the front, an important aspect of the dynamics is the evolution of the cloak thickness. Two limiting regimes can be identified. In the “fast cloaking” regime, transport along the drop surface is rapid compared to transport within the brush, leading to a thin layer that quickly covers the entire drop before thickening. In the opposite “slow cloaking” regime, transport along the surface is rate-limiting, such that the cloak locally reaches its equilibrium thickness before advancing further. Figure 6a shows qualitative expectations for the time evolution of the thickness of the cloak in each of the two extremes. In the fast cloaking regime, we expect the thickness to remain small and approximately constant during spreading and increase only after the cloak reaches the apex of the drop. In contrast, in the slow cloaking regime, the thickness should grow rapidly at early times and subsequently saturate once its local equilibrium value is reached.

To determine which regime applies in our system, we compute the time evolution of the cloak thickness as described in Sec. S.5.4. The results are shown in Fig. 6b for different swelling ratios. The black markers indicate the time at which the cloak front reaches the apex of the drop, defined as the point where the front lies within $3^\circ$ of the top. The observed behavior does not conform to either limiting case. Instead, the cloak thickness increases continuously both before and after the front reaches the apex, indicating that neither surface transport nor diffusion within the brush is overwhelmingly dominant. This places the system in an intermediate regime, where the characteristic timescales for transport along the drop surface and within the brush are comparable. Such a balance of timescales leads to simultaneous spreading and thickening of the cloaking layer.

### Material transport during growth

Having characterized the kinetics of wetting ridge growth and cloaking, we now turn to the underlying material transport within the brush. In particular, we focus on how oil is redistributed during the approach to equilibrium and how this redistribution governs the observed dynamics. Our simulation results motivate the development of a theoretical framework for oil transport that captures the kinetics of ridge growth.

#### Oil depletion

The results of the previous section demonstrate that the growth rate of both the wetting ridge and the cloaking layer depends on the swelling ratio of the brush. This dependence reflects the local availability of oil near the contact line. As the ridge grows, it continuously draws in material from its surroundings, leading to depletion of oil in adjacent regions. Consequently, further growth requires transport of oil from increasingly distant parts of the brush. To quantify this redistribution, we introduce the integrated oil density

24 $$\begin{aligned} \Lambda (\rho ) = \int _{{0}}^{{L_z}} \varrho _{p}(\rho , z) dz, \end{aligned}$$

which represents an effective area density of oil at radial position ρ, averaged over the azimuthal direction.

**Fig. 7:**
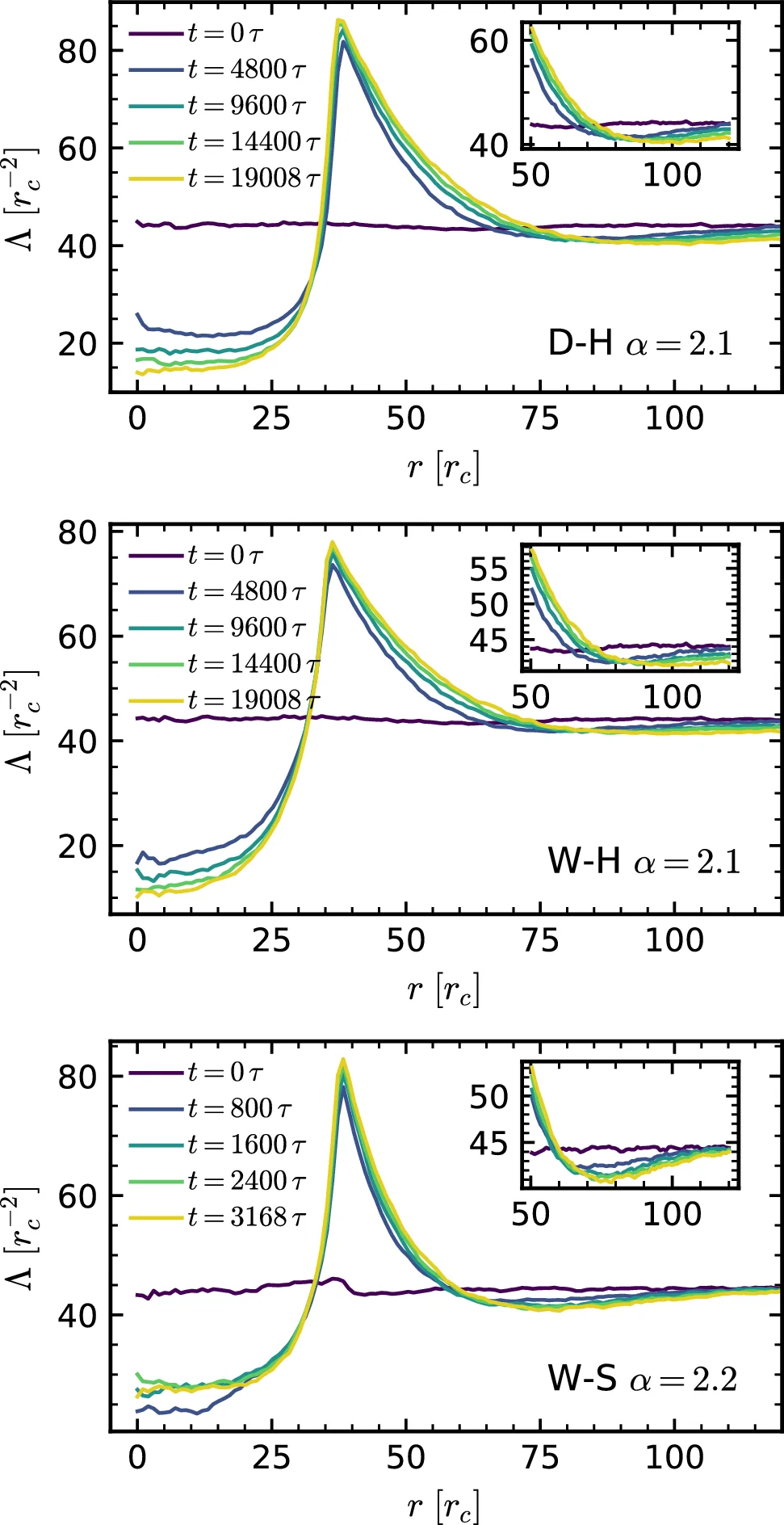
$\Lambda (\rho )$ at different time points during cloaking for the D-H, W-H, and W-S systems. Insets are close ups near the three-phase contact line. There is a clear depletion zone of material outside of the drop. The amount of material also drops from under the droplet

Figure 7 shows the radial profiles $\Lambda (\rho )$ for the different systems at several time points during the simulation; the insets provide a magnified view of the region outside of the drop. Initially, the profile is approximately uniform, reflecting the homogeneous state of the unperturbed brush. As the system evolves, the presence of the drop induces the growth of the wetting ridge in the vicinity of the contact line, which is at a radius $35 \lesssim R_{\textrm{cl}} \lesssim 40 \, [r_c]$ depending on the system. For the W-S system, the presence of the cloak will slightly affect the value of Λ(r) where the drop is present. However, since the thickness of the cloak is much smaller than the thickness of the brush under the drop (see our previous work Ref. 27), we do not expect this contribution to affect the conclusions we draw from our Λ(r) profiles.

Beneath the drop ($r < R_{\textrm{cl}}$), a pronounced decrease in $\Lambda (\rho )$ is observed. This reduction arises from two distinct mechanisms. First, oil is depleted as it is transported toward the contact line and incorporated into the growing ridge. Second, the Laplace pressure of the drop exerts a downward stress on the brush, compressing the grafted chains and expelling oil from the underlying region. At the contact line ($r \approx R_{\textrm{cl}})$) $\Lambda (\rho )$ exhibits a pronounced peak, corresponding to the accumulation of oil in the wetting ridge. Moving further outward ($r>R_{\textrm{cl}}$), a shallow minimum develops, indicating the formation of a depletion zone for polymers outside the drop. At low swelling ratios, this depletion remains localized near the ridge (see Figure S.9). For large swelling ratios, the depletion zone extends over substantial distances and in some cases reaches the boundaries of the simulation domain. Ideally, one should simulate larger boxes; however, this was not possible due to computational limitations.

#### Modeling the material transport

The presence of depletion zones both beneath and outside the drop indicates that oil is removed from the brush faster than it can be replenished locally. As a result, continued growth of the ridge relies on the transport of oil from distant regions. This observation highlights the importance of long-range diffusion within the brush and suggests that the overall kinetics are limited by the rate at which oil can be supplied to the contact line. These findings motivate the development of a continuum description of the transport process. We adapt a diffusion-based model introduced in previous work [49] to the case of polymer brushes and extend the domain to both sides of the wetting ridge, i.e., under and outside of the drop. In addition, we use a variable interaction function in the free energy that depends on the swelling of the brush. Details of the model can be found in section S.4 with the main elements introduced in Sect. 2.3.

To assess the validity of the continuum model, we compare its predictions for the line density of oil at the contact line with the corresponding results from MD simulations of the W-S system. In simulations, the line density is computed as

25 $$\begin{aligned} \lambda ^{\textrm{sim}}(t) = N(t)/2\pi R_{\textrm{cl}}, \end{aligned}$$

where *N*(*t*) is s the total amount of oil contained in the ridge and cloak region, obtained from the density field as

26 $$\begin{aligned} N(t) = 2\pi R_{\textrm{D}}^2 \int _{\theta _{\text {tip}}(t)}^{\theta _{\textrm{max}}} \int _{0}^{R_{\textrm{max}}} \varrho _{o} \sin \theta \, \text {d}r \, \text {d}\theta . \end{aligned}$$

Here $R_{\textrm{D}}$ is the radius of curvature of the drop, $R_{\textrm{cl}}$ is the contact line radius, and $\theta _{\textrm{max}} = 75^\circ$. The free parameters of the model, *M* and $\mathcal {B}$, are determined by adjusting the theoretical prediction to the simulations at a reference swelling ratio α=2.2. This procedure yields

27 $$\begin{aligned} M = 10 ~~~ ; ~~~ \mathcal {B} = 0.08. \end{aligned}$$

Keeping these parameters fixed, we then vary the swelling ratio and compute the corresponding time evolution of the line density at the contact line.

**Fig. 8:**
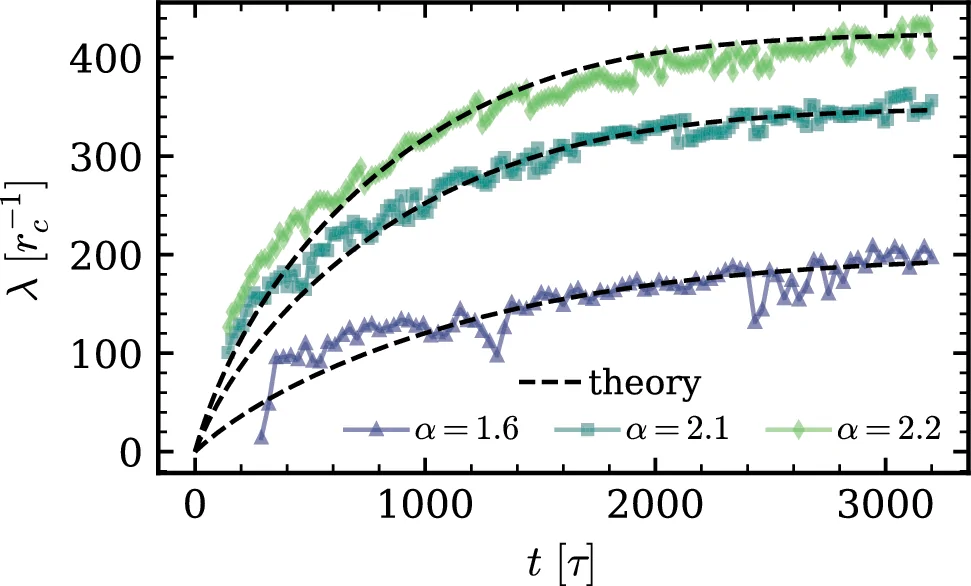
The line density at the ridge from simulation (data points) and theory (dashed lines)

The comparison between model and simulation is shown in Fig. 8. Overall, the model captures the temporal evolution of the line density well at intermediate and long times for all swelling ratios considered. In particular, it reproduces both the growth rate and the saturation behavior observed in the simulations. At early times, systematic deviations are observed, with the simulations exhibiting a more rapid initial increase in λ than predicted by the model. This discrepancy can likely be attributed to advective effects associated with the initial formation of the wetting ridge, during which material is rapidly drawn toward the contact line. Such processes are not included in the present description, which assumes purely diffusive transport within the brush.

As discussed above, the free energy functional includes a concentration-dependent interaction term through $\chi (\varphi )$. To assess its impact, we also solve the model with this term omitted, while recalibrating the parameters κ and $\mathcal {B}$. The resulting predictions (see Fig. S.10) are found to be very similar to those obtained with the full model. This indicates that, within the parameter range explored here, the detailed form of the interaction term plays only a minor role in determining the overall kinetics.

## Conclusion

We have investigated the kinetics of wetting ridge growth and droplet cloaking on lubricant-infused polymer brushes using a combined experimental, simulation, and theoretical approach. Our analysis suggests different dynamical regimes governed by the interplay between interfacial forces, brush elasticity, and the transport of lubricant within the brush.

We identify distinct regimes of ridge growth and cloaking that depend sensitively on the swelling state of the brush and the sign of the spreading parameter. While a positive spreading parameter favors cloaking at equilibrium, our results highlight that the transient dynamics can be complex, with the evolution of the system controlled by the availability and redistribution of oil within the brush. For negative spreading parameter, cloaking is absent, but substantial restructuring of the lubricant phase may still occur near the contact line.

A central finding of this work is the strong coupling between ridge growth and oil transport. The formation of the wetting ridge is accompanied by pronounced depletion of oil both beneath and outside the drop, indicating that local supply is insufficient to sustain growth. As a result, the evolution of the system is governed by transport from increasingly distant regions of the brush. Moreover we show that, above a critical swelling, the lubricant can locally separate from the brush within the ridge. This phase separation provides an additional pathway for lubricant depletion and is therefore likely to play an important role in determining the long-term stability of lubricant-infused surfaces.

To rationalize these observations, we developed a continuum diffusion model based on the free energy of the brush and its coupling to the contact line. Despite its simplicity, the model quantitatively captures the evolution of the oil line density at intermediate and late times across a range of swelling ratios. This agreement indicates that the kinetics of ridge growth and cloaking are largely controlled by diffusive transport of oil within the brush. Deviations at early times can be attributed to advective processes associated with the initial formation of the ridge, which are not included in the present description.

Our results also provide insight into the dynamics of cloaking on droplets. In particular, we find that the propagation of the cloaking front and the thickening of the film can occur on comparable time scales, reflecting a balance between transport along the drop interface and within the brush. This highlights the inherently coupled nature of surface and bulk transport processes in these systems.

Our results can be viewed in the context of previous work on lubricant-infused surfaces [29], where the growth of the wetting ridge was identified as the primary source of lubricant depletion and its dynamics were rationalized in terms of pressure gradients and viscous stresses in a porous substrate. The present study extends this framework to polymer brushes, where the lubricant is stored within a deformable medium and transport is governed by a combination of diffusion and thermodynamic driving forces. In this setting, we further identify local phase separation within the ridge as an additional pathway for lubricant depletion, which is not captured in models of non-deformable substrates.

Overall, our results demonstrate that the dynamics of wetting ridge growth and cloaking on polymer brushes can be understood within a unified framework that combines interfacial thermodynamics with diffusion-limited transport. These insights extend existing concepts for lubricant-infused surfaces to soft, deformable substrates and provide guidance for the design of coatings with improved resistance to depletion and enhanced long-term performance.

## Supplementary Information

Below is the link to the electronic supplementary material.

**Supplementary information** Supplementary Information is available with a table containing the values of physical parameters in simulation such as surface tension and viscosity, a description of the brush free energy with varying interaction term and the method to determine that term, a derivation of the diffusion equation used to model the material transport, a description of some of the methods used in the simulations and subsequent analysis, additional data on the oil separation in the W-H system, simulation snapshots of contacting drops of liquid and oil to illustrate the presence or absence of cloaking, additional graphs for the non-equilibrium distribution of oil during ridge growth and cloaking, and a figure comparing the performance of the theoretical model with a varying interaction function to the one where interactions are absent. (pdf 12,108 KB)

## Data Availability

All codes used for simulation are available on the github repository https://github.com/rodbadr/dropletOnLubricatedBrush-HOOMD4. Data used for the figures along with plotting codes, as well as the codes used to solve the diffusion equation, are made available on the Zenodo repository https://doi.org/10.5281/zenodo.19557302. The exception is the data for the figures that require the density maps due to file size limitations. Such data can be made available upon reasonable request.
