# Supplementary material for: Kinetics of droplet cloaking and wetting ridge growth on lubricated polymer brushes
Source: Eur Phys J E Soft Matter. 2026 Jul 20;49(8):62. doi: 10.1140/epje/s10189-026-00607-6 (PMC13381383; doi:10.1140/epje/s10189-026-00607-6)
Supplement: Supplementary file 1 — Supplementary information Supplementary Information is available with a table containing the values of physical parameters in simulation such as surface tension and viscosity, a description of the brush free energy with varying interaction term and the method to determine that term, a derivation of the diffusion equation used to model the material transport, a description of some of the methods used in the simulations and subsequent analysis, additional data on the oil separation in the W-H system, simulation snapshots of contacting drops of liquid and oil to illustrate the presence or absence of cloaking, additional graphs for the non-equilibrium distribution of oil during ridge growth and cloaking, and a figure comparing the performance of the theoretical model with a varying interaction function to the one where interactions are absent. (pdf 12,108 KB) [file 10189_2026_607_MOESM1_ESM.pdf]

Supplementary Information

S.1 Simulation parameters and physical quantities

	ρ_p	ρ_l	γ_p	γ_l	γ_{pl}	$S_{p/l}$	θ_Y	μ_p	μ_l
W-S	2.9	4	0.841(20)	3.14(4)	1.41(3)	0.88(6)	100.5(7)	5.171(27)	5.806(8)
W-H	3.3	4.2	1.445(14)	3.74(4)	2.728(20)	−0.43(6)	110.1(5)	9.12(7)	6.785(4)
D-H	3.3	4	1.49(3)	3.15(3)	1.54(5)	0.12(4)	91(1)	9.12(7)	5.806(8)

Table S.1: Results for the equilibrium densities, interfacial tension values, the spreading parameter of the oil on the liquid drop $S_{p/l}$, the estimated Young contact angle, and the viscosities. W-S is the Water-Silicone oil system, W-H is Water-Hexadecane, and D-H is DMSO40-Hexadecane. A subscript 'p' corresponds to polymer, while 'l' corresponds to liquid, and two letters correspond to interface between the two species. All values are in simulation units set by $r_c = 1$; $k_B T = 1$; $m = 1$. Values in parentheses are uncertainties in the rightmost digits.

S.2 Neumann balance

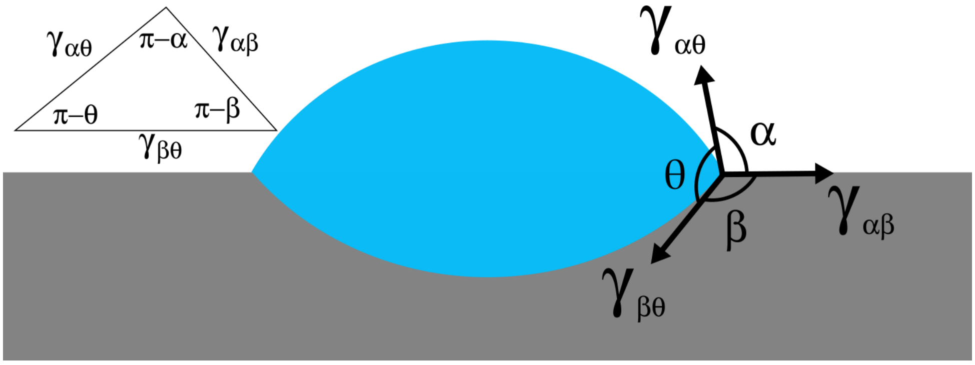

Fig. S.1: Cartoon of a drop on a liquid surface. The configuration is that of a liquid lens, with three angle α, β, and θ at the three phase contact line. The angles are set by the balance of the three surface tensions between the three phases. Also shown is the corresponding Neumann triangle for this configuration. Reproduced from [50] under CC-BY-SA 4.0.

For two immiscible liquids in contact, at the three phase contact line, the equilibrium is dictated by the Neumann balance of forces. We label the three fluid phases as α, β, and θ, which also corresponds to the angle in each phase. The surface tensions are labeled γ_{XY} for the tension at the interface between phases X and Y, with $X,\ Y \in \{\alpha, \beta, \theta\}$. The liquid surface is fully deformable and the equilibrium configuration is that of a liquid lens. Balancing forces (per unit length) in both the vertical and horizontal directions yields

$$\begin{aligned} \gamma_{\alpha\theta} + \gamma_{\beta\theta}\cos\theta + \gamma_{\alpha\beta}\cos\alpha &= 0 \\ \gamma_{\alpha\theta}\cos\theta + \gamma_{\beta\theta} + \gamma_{\alpha\beta}\cos\beta &= 0 \\ \gamma_{\alpha\theta}\cos\alpha + \gamma_{\beta\theta}\cos\beta + \gamma_{\alpha\beta} &= 0. \end{aligned} \tag{S.1}$$

The configuration satisfying the equations above is known as a Neumann configuration. Another way of identifying the angles in a Neumann configuration is through what is known as a Neumann triangle. One can construct a triangle whose side lengths are proportional to the surface tensions between the three phases as shown on the left side of Figure S.1. The angles in the triangle can be mapped to the contact angles in the wetting configuration. We can see from this analogy that a Neumann configuration is only possible if the surface tensions satisfy a triangle inequality

$$\gamma_{XY} + \gamma_{YZ} \geq \gamma_{XZ} \tag{S.2}$$

with $X, Y, Z \in \{\alpha, \beta, \theta\}$. The inequality needs to be satisfied for all permutations of α, β, and θ. If the triangle inequality is broken, the equilibrium configuration is either one where the deposited liquid spreads on the surface, is engulfed by it, or fully detaches from it, depending on the spreading parameters. The Neumann triangle view allows us then to write practical equations for the angles in terms of the surface tensions using the law of cosines

$$\cos X = \frac{\gamma_{YZ}^2 - \gamma_{XY}^2 - \gamma_{XZ}^2}{2\gamma_{XY}\gamma_{XZ}} \tag{S.3}$$

where again $X, Y, Z \in \{\alpha, \beta, \theta\}$.

S.3 Variable interaction function

S.3.1 Free energy with constant interaction parameter

Notation:

- n_o: number of oil molecules
- N_o: length of an oil molecule in number of monomers
- n_B: number of grafted chains
- N_B: length of a grafted chain in number of monomers
- φ: number fraction of oil monomers in relation to grafted chain monomers
- k: elasticity of the brush assuming it deviates from the ideal value 3
- H_B: thickness of the brush layer
- μ_B: chemical potential in the brush
- σ: grafting density of the brush
- a: size of a monomer

The free energy of the brush with an arbitrary solvent can be written as [27, 48]

$$\frac{\mathcal{F}}{k_B T} = n_o \ln \varphi + \chi n_o (1 - \varphi) + \frac{k}{2} \frac{n_B}{N_B} H_B^2 - n_o \mu_B \tag{S.4}$$

where the symbols are defined in the notation above. The reference free energy is that of a saturated brush so that the chemical potential vanishes at saturation. The number fraction of oil φ and that of grafted chain monomers $1 - \varphi$ are given by

$$\varphi = \frac{n_o N_o}{n_o N_o + n_B N_B} \tag{S.5}$$

$$1 - \varphi = \frac{n_B N_B}{n_o N_o + n_B N_B} \tag{S.6}$$

The height of the brush H_B is given by

$$H_B = \frac{N_B \sigma a^3}{1-\varphi}. \tag{S.7}$$

From Eq. S.4 we write the free energy per grafted chain monomer $\tilde{\mathcal{F}} = \mathcal{F}/(n_B N_B)$, also substituting Eq. S.7 and setting $a = 1$

$$\frac{\tilde{\mathcal{F}}}{k_B T} = \frac{n_o}{n_B N_B} \ln \varphi + \chi \frac{n_o}{n_B N_B}(1-\varphi) + \frac{k}{2}\frac{\sigma^2}{(1-\varphi)^2} - \frac{n_o}{n_B N_B}\mu_B. \tag{S.8}$$

Using Eq. S.5 and S.6 we can write

$$\frac{n_o}{n_B N_B} = \frac{\varphi}{N_o}\frac{n_o N_o + n_B N_B}{n_B N_B} \tag{S.9}$$

$$= \frac{1}{N_o}\frac{\varphi}{1-\varphi} \tag{S.10}$$

which we substitute into Eq. S.8 to get

$$\frac{\tilde{\mathcal{F}}}{k_B T} = \frac{1}{N_o}\frac{\varphi}{1-\varphi}\ln\varphi + \chi\frac{1}{N_o}\varphi + \frac{k}{2}\frac{\sigma^2}{(1-\varphi)^2} - \frac{1}{N_o}\frac{\varphi}{1-\varphi}\mu_B \tag{S.11}$$

$$= \tilde{\mathcal{F}}_0 + \chi\frac{1}{N_o}\varphi \tag{S.12}$$

where $\tilde{\mathcal{F}}_0$ includes the terms that do not depend on the interaction parameter.

S.3.2 Free energy with variable interaction parameter

Following Refs. 47 and 48, we can modify our free energy Eq. S.12 by making the interaction parameter depend on the fraction of oil in the brush. The equilibrium is obtained by setting $\partial\tilde{\mathcal{F}}/\partial\varphi = 0$

$$\frac{\partial\tilde{\mathcal{F}}}{\partial\varphi} = \frac{\partial\tilde{\mathcal{F}}_0(\varphi,\mu)}{\partial\varphi} + \frac{1}{N_o}\left[\chi(\varphi) + \varphi\chi'(\varphi)\right] = 0. \tag{S.13}$$

The differential equation can be solved for $\chi(\varphi)$ and we finally get (see Ref. 48 SI)

$$\chi(\varphi) = \frac{N_o}{\varphi}\left(\tilde{\mathcal{F}}_0(\varphi^*, 0) - \tilde{\mathcal{F}}_0(\varphi, \mu)\right) \tag{S.14}$$

where φ^* is the oil fraction at saturation. It is worth noting here that our Eq. S.14 is different from that in Ref. 48 as we choose the saturated brush as a reference when solving the differential equation, leading to a vanishing interaction function at saturation. The motivation for this is that the lubricant chains are much shorter than the grafted chains. Therefore, since the two are chemically identical, we expect the lubricant to act as an athermal solvent for the brush after the brush is saturated.

S.4 Diffusion model derivation

In the following, we assume that our system has azimuthal symmetry; in addition, we assume that the vertical advection in the brush is much faster than the lateral diffusion so that the concentration profile is homogeneous along the z-axis. The local concentration profile then only depends on the radial position ρ.

S.4.1 Free energy functional

To derive our dynamic equations, we need an expression for a free energy that depends on the local fraction of oil. We assume a functional form as the integral of a free energy density $f_B(\varphi(x))$

$$\frac{\mathcal{F}_B[\varphi(x)]}{k_B T} = \int \mathrm{d}V\, f_B(\varphi(x)). \tag{S.15}$$

The free energy density of the brush can be written as

$$f_B(\varphi(x)) = \frac{\varphi}{N_o} \ln \varphi + \frac{\chi(\varphi)}{N_o} \varphi\,(1-\varphi) + \frac{k}{2} \frac{\sigma^2}{1-\varphi}. \tag{S.16}$$

One can easily verify that integrating this expression over the volume of the brush with a uniform oil fraction φ will result in the correct brush free energy Eq. S.4 but in the canonical ensemble instead of grand canonical. To do this we use Eq. S.7 and (with $a = 1$)

$$\varphi V = n_o N_o \tag{S.17}$$

$$\int \mathrm{d}V \frac{k}{2} \frac{\sigma^2}{1-\varphi} = A \times H_B \frac{k}{2} \frac{\sigma^2}{1-\varphi} \tag{S.18}$$

$$\sigma A = n_B \tag{S.19}$$

where V and A are the volume and area of the brush respectively. At equilibrium and for a given oil fraction φ^{eq}, the chemical potential of the brush is

$$\mu_B^{\mathrm{eq}} = \frac{\partial \mathcal{F}_B}{\partial n_o} = \ln \varphi^{\mathrm{eq}} + 1 - \varphi^{\mathrm{eq}} + (1-\varphi^{\mathrm{eq}})^2 \left(\chi + \chi' \varphi^{\mathrm{eq}}\right) + k \frac{\sigma^2 N_o}{1-\varphi^{\mathrm{eq}}} \tag{S.20}$$

where we used

$$\frac{\partial \varphi}{\partial n_o} = \frac{\varphi}{n_o}(1-\varphi). \tag{S.21}$$

In addition to the free energy of the brush, we account for the contribution of the three phase contact line through a free energy line-density $f_{\mathrm{cl}}(\lambda)$ where λ is the line density of oil at the three phase contact line. With this, the free energy of the contact line $\mathcal{F}_{\mathrm{cl}}$ is given by

$$\frac{\mathcal{F}_{\mathrm{cl}}[\lambda]}{k_B T} = \oint \mathrm{d}l\, f_{\mathrm{cl}}(\lambda) \tag{S.22}$$

where the integral is evaluated around the contact line. We assume the free energy line-density to be quadratic in λ and with a stiffness κ

$$f_{\mathrm{cl}}(\lambda) = \frac{\kappa}{2} (\lambda - \lambda_0)^2 \tag{S.23}$$

where λ_0 is the saturation line-density of the contact line. The chemical potential for the contact line is

$$\mu_{\mathrm{cl}} = \frac{\partial f_{\mathrm{cl}}}{\partial \lambda} = \kappa(\lambda - \lambda_0). \tag{S.24}$$

The total free energy of the system can the be written as

$$\frac{\mathcal{F}[\varphi(x), \lambda]}{k_B T} = \int \mathrm{d}V \left\{ \frac{\varphi}{N_o} \ln \varphi + \frac{\chi(\varphi)}{N_o} \varphi\,(1-\varphi) + \frac{k}{2} \frac{\sigma^2}{1-\varphi} \right\} + \oint \mathrm{d}l\, \kappa\, (\lambda - \lambda_0)^2 \tag{S.25}$$

S.4.2 Dynamical Equations

S.4.2.1 Evolution of the line density

At the contact line, we assume that the rate of change of the line density of oil λ is proportional to the difference in chemical potential and to the amount of lubricant in the brush at the location of the three phase contact line.

$$\frac{\partial \lambda}{\partial t} = -\mathcal{B}\Phi(R_{\mathrm{cl}})\left[f'(\lambda) - \mu_B(R_{\mathrm{cl}}, t)\right] \tag{S.26}$$

where $\mathcal{B}$ is a filling rate, $\Phi(\rho) = \int_0^{H_B} \varphi(\rho, z)\,\mathrm{d}z$, R_{cl} is the radial position of the contact line, and $\mu_B(\rho, t)$ is a locally defined chemical potential for the brush. To simplify the picture, we assume that the chemical potential in the brush is always close to its equilibrium value from Eq. S.20. Therefore, we rewrite the evolution equation Eq. S.26 for λ as

$$\frac{\partial \lambda}{\partial t} = -\mathcal{B}\Phi(R_{\mathrm{cl}})\kappa\left(\lambda - \lambda_e\right) \tag{S.27}$$

$$\lambda_e = \lambda_0 + \frac{\mu_B^{\mathrm{eq}}}{\kappa} \tag{S.28}$$

where we define the equilibrium line density $\lambda_e(\varphi^{\mathrm{eq}})$ for a given equilibrium fraction of oil in the brush

S.4.2.2 Evolution of the concentration profile

We split our full domain into two regions separated by the contact line at $\rho = R_{\mathrm{cl}}$. In each region we define a current $j_\rho^<$ for the region $\rho < R_{\mathrm{cl}}$ and $j_\rho^>$ for $\rho > R_{\mathrm{cl}}$. At any position ρ, the current in the radial direction can be written as

$$j_\rho = -M\varphi\nabla_\rho \frac{\delta \mathcal{F}_B\left[\varphi\right]}{\delta\varphi} \tag{S.29}$$

where M is the mobility of oil in the brush, $\nabla_\rho = \partial/\partial\rho$ is the radial component of the gradient operator in cylindrical coordinates, and $\delta/\delta\varphi$ denotes the variational derivative with respect to the concentration profile $\varphi(\rho)$. From the current, the evolution equation for the profile can be obtained as the continuity equation

$$\frac{\partial \varphi}{\partial t} = -\nabla_\rho j_\rho. \tag{S.30}$$

Using Eq. 9 we can evaluate the variational derivative of our free energy functional

$$\begin{aligned}\frac{\delta \mathcal{F}_B\left[\varphi\right]}{\delta\varphi} &= \frac{\partial f_B}{\partial \varphi}\\ &= \frac{\ln\varphi}{N_o} + \frac{1}{N_o} + \frac{\chi'}{N_o}\varphi\left(1-\varphi\right) + \frac{\chi}{N_o}\left(1-2\varphi\right) + \frac{k}{2}\frac{\sigma^2}{\left(1-\varphi\right)^2}\end{aligned} \tag{S.31}$$

and subsequently calculate the gradient

$$\nabla_\rho \frac{\delta \mathcal{F}_B\left[\varphi\right]}{\delta\varphi} = \left\{\frac{1}{N_o\varphi} + k\frac{\sigma^2}{\left(1-\varphi\right)^3} + \frac{1}{N_o}\left[\chi''\varphi\left(1-\varphi\right) + 2\chi'\left(1-2\varphi\right) - 2\chi\right]\right\}\nabla_\rho\varphi \tag{S.32}$$

which eventually gives the expression for the radial current

$$j_\rho = -M\varphi \left\{ \frac{1}{N_o\varphi} + k\frac{\sigma^2}{(1-\varphi)^3} + \frac{1}{N_o}\left[\chi''\varphi(1-\varphi) + 2\chi'(1-2\varphi) - 2\chi\right] \right\} \nabla_\rho \varphi \tag{S.33}$$

$$= -M \left\{ \frac{1}{N_o} + k\frac{\sigma^2\varphi}{(1-\varphi)^3} + \frac{\varphi}{N_o}\left[\chi''\varphi(1-\varphi) + 2\chi'(1-2\varphi) - 2\chi\right] \right\} \nabla_\rho \varphi \tag{S.34}$$

The final evolution equation for the concentration profile is therefore

$$\frac{\partial\varphi}{\partial t} = M \Bigg\{ \left[\frac{1}{N_o} + k\frac{\sigma^2\varphi}{(1-\varphi)^3} + \frac{\varphi}{N_o}\left[\chi''\varphi(1-\varphi) + 2\chi'(1-2\varphi) - 2\chi\right] \right] \nabla_\rho^2 \varphi + \left[k\sigma^2 \frac{1+2\varphi}{(1-\varphi)^4} + \frac{1}{N_o}\left(\chi^{(3)}\varphi^2(1-\varphi) + \chi''\varphi(4-7\varphi) + \chi'(2-10\varphi) - 2\chi \right) \right] (\nabla_\rho\varphi)^2 \Bigg\} \tag{S.35}$$

To simplify the notation, we rewrite the current and the evolution in more compact forms. For the current we have

$$j_\rho(\varphi(t)) = -M\left[j_\rho^0(\varphi(t)) + j_\rho^{\text{int}}(\varphi(t))\right] \nabla_\rho\varphi \tag{S.36}$$

$$j_\rho^0(\varphi(t)) = \frac{1}{N_o} + k\frac{\sigma^2\varphi}{(1-\varphi)^3} \tag{S.37}$$

$$j_\rho^{\text{int}}(\varphi(t)) = \frac{\varphi}{N_o}\left[\chi''\varphi(1-\varphi) + 2\chi'(1-2\varphi) - 2\chi\right] \tag{S.38}$$

and for the evolution equation we have

$$\frac{\partial\varphi}{\partial t} = M\left[g_1(\varphi(t))\,(\nabla_\rho\varphi)^2 + g_2(\varphi(t))\nabla_\rho^2\varphi\right] \tag{S.39}$$

$$g_1(\varphi(t)) = g_1^0(\varphi(t)) + g_1^{\text{int}}(\varphi(t)) \tag{S.40}$$

$$g_1^0(\varphi(t)) = k\sigma^2\frac{1+2\varphi}{(1-\varphi)^4} \tag{S.41}$$

$$g_1^{\text{int}}(\varphi(t)) = \frac{1}{N_o}\left(\chi^{(3)}\varphi^2(1-\varphi) + \chi''\varphi(4-7\varphi) + \chi'(2-10\varphi) - 2\chi\right) \tag{S.42}$$

$$g_2(\varphi(t)) = g_2^0(\varphi(t)) + g_2^{\text{int}}(\varphi(t)) \tag{S.43}$$

$$g_2^0(\varphi(t)) = j_\rho^0(\varphi(t)) = \frac{1}{N_o} + k\frac{\sigma^2\varphi}{(1-\varphi)^3} \tag{S.44}$$

$$g_2^{\text{int}}(\varphi(t)) = j_\rho^{\text{int}}(\varphi(t)) = \frac{\varphi}{N_o}\left[\chi''\varphi(1-\varphi) + 2\chi'(1-2\varphi) - 2\chi\right] \tag{S.45}$$

S.4.2.3 Boundary conditions

To solve the evolution equations we need to set the appropriate boundary conditions at $\rho = 0$, $\rho = R_{\text{cl}}$, and $\rho = L$ with L the length of the domain.

At $\rho = 0$, the azimuthal symmetry of our system implies

$$j_\rho^<(0,t) = 0 \tag{S.46}$$

since there is no preferred flow direction at the axis of symmetry.
At $\rho = L$, we consider the brush to be in contact with an infinitely larger reservoir of oil that maintains the fraction at the edge to a preset value φ_B

$$\varphi(L,t) = \varphi_B. \tag{S.47}$$

Finally, at the contact line $\rho = R_{\mathrm{cl}}$, we require that all of the oil leaving the brush will be incorporated into the ridge/cloak. This means that the rate of change of λ must match the total current coming from both sides of the contact line, which we write as

$$\frac{\partial \lambda}{\partial t} = -H(\varphi(R_{\mathrm{cl}}))\left(j_\rho^>(R_{\mathrm{cl}}) - j_\rho^<(R_{\mathrm{cl}})\right) \tag{S.48}$$

S.4.3 Numerical implementation

In order to solve the equation numerically, we split the domain radially into bins with width Δr, and label the separate bins as r_i with $r_0 = R$. For any function $g(r)$ we call $g_i \equiv g(r_i)$. We use the discretization scheme :

$$\frac{\partial g_i}{\partial r} = \frac{1}{2\Delta r}(g_{i+1} - g_{i-1}) \tag{S.49}$$

$$\frac{\partial^2 g_i}{\partial r^2} = \frac{1}{\Delta r^2}(g_{i+1} + g_{i-1} - 2g_i) \tag{S.50}$$

and the forward steps in time are executed as a forward Euler scheme.

The boundary condition at the origin is imposed by setting

$$\varphi_0(t) = \varphi_1(t) \tag{S.51}$$

while the boundary condition at the edge of the domain is imposed by maintaining for $\varphi(L,t) \equiv \varphi_L(t)$

$$\varphi_L(t) = \varphi_B \tag{S.52}$$

The boundary condition at the contact line Eq. S.48 is not as straightforward. The current on either side of the contact line is given by Eqs. S.36-S.38. However, to evaluate the derivative that appears there numerically, we use the backward and forward two-point formulas for the inner and outer domain respectively

$$\nabla_\rho^< \varphi|_{\rho=R_{\mathrm{cl}}} = \frac{\varphi(R_{\mathrm{cl}}) - \varphi(R_{\mathrm{cl}} - \Delta\rho)}{\Delta\rho} \tag{S.53}$$

$$\nabla_\rho^> \varphi|_{\rho=R_{\mathrm{cl}}} = \frac{\varphi(R_{\mathrm{cl}} + \Delta\rho) - \varphi(R_{\mathrm{cl}})}{\Delta\rho}. \tag{S.54}$$

Using Eq. S.27, S.36, S.53, and S.54, and defining $\varphi(R_{\mathrm{cl}}, t)) \equiv \varphi_{\mathrm{cl}}(t)$ we rewrite eq. S.48 in the following way

$$-\mathcal{B}\Phi\left(R_{\mathrm{cl}}\right)\kappa(\lambda - \lambda_e) = H(R)M\left\{\left[j_\rho^0(\varphi_{\mathrm{cl}}(t)) + j_\rho^{\mathrm{int}}(\varphi_{\mathrm{cl}}(t))\right]\right\} \times \tag{S.55}$$

$$\frac{\varphi(R_{\rm cl} - \Delta\rho) - 2\varphi_{\rm cl} + \varphi(R_{\rm cl} + \Delta\rho)}{\Delta\rho}.$$

Given the value of φ at either side of the contact line, we can solve this equation for $\varphi_{\rm cl}$. In particular, we assume that our brush is thin enough for the concentration profile to be independent of z, so that $\Phi(\rho) = H(\rho)\varphi(\rho)$, and our boundary condition equation becomes

$$-\mathcal{B}\varphi_{\rm cl}\kappa(\lambda - \lambda_e) = M\left[j_\rho^0(\varphi_{\rm cl}(t)) + j_\rho^{\rm int}(\varphi_{\rm cl}(t))\right] \times \frac{\varphi(R_{\rm cl} - \Delta\rho) - 2\varphi_{\rm cl} + \varphi(R_{\rm cl} + \Delta\rho)}{\Delta\rho}. \quad \text{(S.56)}$$

S.5 Methods

S.5.1 System preparation

S.5.1.1 Liquid slab preparation

Our systems of interest require the preparation of a liquid coexisting with a vapor phase in a slab geometry. The slab can consist of either a simple liquid or a polymeric liquid. We refer to the latter as a melt. The slab is prepared in two stages. In the first stage, chains or individual particles are equilibrated in a box with periodic boundary conditions. The size of the box in the $x - y$ direction is chosen manually. The size in the z-direction is chosen so that the number density of monomers has a specific value. If the density of the liquid phase at coexistence is known, the starting density is chosen close to that. From experience, it is better to choose the starting density as slightly smaller than the coexistence value. In the second stage the size of the simulation box in the z-direction is doubled. When simulating a polymeric liquid, chains that cross the boundary in the original box are properly unwrapped. The system is then left to equilibrate until a single liquid slab is coexisting with a dilute vapor phase.

S.5.1.2 Dry brush preparation

The polymer brush consists of chains of length N_B grafted by one end by fixing the end monomer to one position. The position of the grafted monomer coincides with the purely repulsive flat surface. The brush has $n_B = N_x \times N_y$ chains, with N_x and N_y the number of grafting sites in the x and y directions respectively. The grafting sites are distributed on a square lattice with a lattice constant (distance between nearest sites) equal to d; this results in a grafting density

$$\sigma = \frac{1}{d^2}. \quad \text{(S.57)}$$

The polymer brush is initialized with the chains fully elongated along the z-direction, then left to relax until equilibrium is reached. With the choice of parameters described in section 2.1, the brush is in bad solvent and the equilibrium corresponds to a collapsed state with a rough topography.

S.5.1.3 Swollen brush preparation

To swell the brush, it is placed in contact with a melt of oil chains prepared as described in section S.5.1.1. The number of chains in the melt is n_o and each chain has length N_o. The size of the melt in the $x - y$ direction is equal to that of the brush, which results in faster equilibration as the diffusion of free chains is quite slow. In the next step, we place the melt in the vicinity of the brush, and run the simulation until the brush absorbs the oil and swells to its equilibrium thickness. The number fraction $\tilde{\Phi}$ of oil monomers to the total number of monomers is calculated as

$$\tilde{\Phi} = \frac{\text{number of oil monomers}}{\text{total number of monomers}} = \frac{n_o N_o}{n_o N_o + n_B N_B}. \quad \text{(S.58)}$$

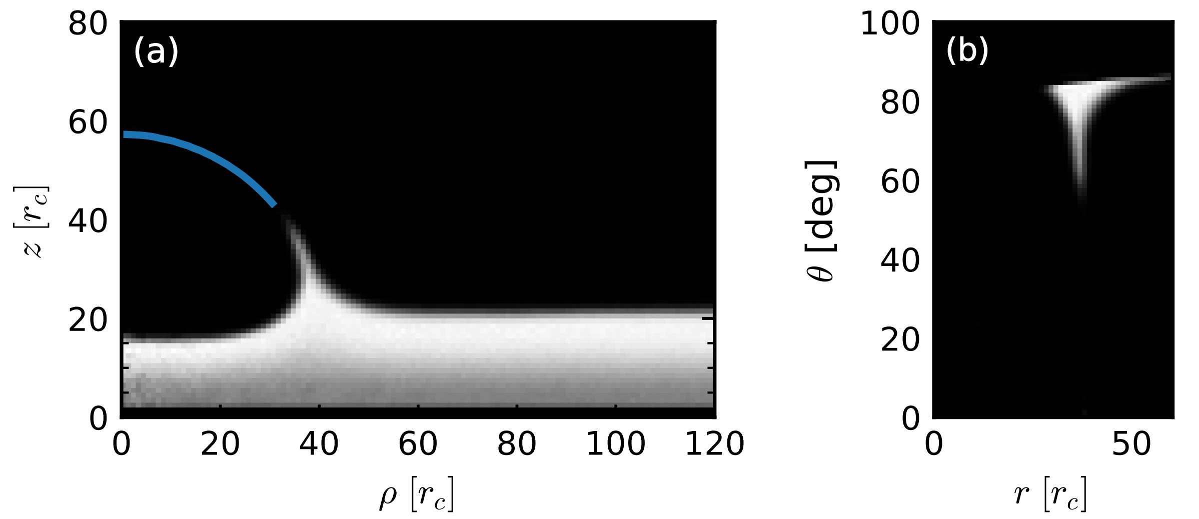

Fig. S.2: (a) Density map of oil in cylindrical coordinates $\varrho_o(\rho, z)$. Blue line indicates the droplet-vapor interface. (b) Density map of oil in spherical coordinates centered at the center of curvature of the drop $\varrho_o(r, \theta)$.

S.5.1.4 Liquid drop preparation and deposition

To prepare a drop to be deposited on the brush, the first step is to prepare a slab of simple liquid as described in section S.5.1.1. The second step is to use this slab to deposit a droplet on the brush. To have good control over the contact radius and size of the droplet, we extract a hemisphere of radius R from the slab and place it in contact with the brush, which is the starting point of our simulations.

S.5.2 Density maps

For a particular simulation snapshot, the first step is to shift the coordinates of all particles so that the center of mass of the droplet coincides with the z-axis, while accounting for periodic boundary conditions. The density map ϱ is created by counting the number of particles belonging to each bin and dividing by the volume of the bin. To avoid confusion, we will stick to the symbol ϱ_x to denote density maps and ρ_x to denote equilibrium bulk densities of species x. We will refer to the radial coordinate in cylindrical coordinates as ρ with no subscript, and in spherical coordinates as r.

S.5.2.1 Cylindrical coordinates

The choice of cylindrical coordinates is motivated by the azimuthal symmetry of the drop. Therefore, the box is partitioned into bins in the z-direction and the radial direction, with respective sizes Δz and $\Delta\rho$. The bin edges are positioned at

$$\rho_i = i \times \Delta\rho \tag{S.59}$$

$$z_j = j \times \Delta z \tag{S.60}$$

with $i, j \in \mathbb{N}$ the indices of the radial bin and of axial bins respectively. In this case, bins have different volumes depending on the index of the radial bin

$$\Delta V_{ij} = \pi \Delta z \Delta\rho^2 (2i + 1). \tag{S.61}$$

An example of such a density map $\varrho_o(\rho, z)$ for the oil is shown in Fig. S.2 (a).

S.5.2.2 Spherical coordinates

The origin of the coordinate system is placed at the center of curvature of the droplet. The box is partitioned into bins in the radial and polar directions, with respective sizes Δr and $\Delta\theta$. The bin edges are positioned at

$$r_i = i \times \Delta r \tag{S.62}$$

$$\theta_j = j \times \Delta\theta \tag{S.63}$$

with $i, j \in \mathbb{N}$ the indices of the radial bin and of polar bins respectively. In this case, bins have different volumes depending on the index of the radial bin

$$\Delta V_{ij} = \frac{2\pi}{3}\Delta r^3(\cos\theta_j - \cos\theta_{j+1})[(i+1)^3 - i^3]. \tag{S.64}$$

An example of such a density map $\varrho_o(r, \theta)$ for the oil is shown in Fig. S.2 (b).

S.5.3 Equal density contours

Having computed the density maps, one key method for delineating the boundaries between the different phases is through equal density contour lines. An example of such a contour is shown in Figure S.2 (a), where the line is an equal density contour $\varrho_l(\rho, \varphi) = \rho_l/2$, with ρ_l the bulk density of the bulk polymer phase. Density contours are used in various ways to extract information from the simulations as described below.

S.5.3.1 Droplet shape

One way of using the density contours is to characterize the shape of the droplet. In the case of a sessile droplet, a density contour at $\varrho_l(\rho, z) = \rho_l/2$ is extracted for the density map of the droplet; afterwards, the top half is fit to a circle, providing us with the coordinates of the center of curvature and the radius of curvature of the droplet. This information is then used to calculate the spherical density maps $\varrho_x(r, \theta)$.

S.5.3.2 Unperturbed brush height

To find the height of the brush far away from the droplet, we calculate density contour $\varrho_o(\rho, z) = \rho_o/2$ from the density map of oil, where ρ_o is the density of the bulk polymer phase. From this contour, we then calculate the average height far away from the drop, and use that as the height of the unperturbed brush. This height is used as the reference to calculate the height of the wetting ridge and the apparent contact angles.

S.5.3.3 Apparent contact angle

Knowing the height of the brush away from the droplet, we can use it as a baseline for calculating apparent contact angles. In our work, the apparent contact angles are always defined as the angle the fit to the droplet shape makes with a horizontal line at the height of the unperturbed brush

$$\theta_{\text{app}} = \frac{\pi}{2} - \arcsin\left(\frac{z_B - z_c}{R_{\text{D}}}\right) \tag{S.65}$$

where z_B is the z-coordinate of the surface of the unperturbed brush, z_c is the z-coordinate of the center of curvature of the droplet, and R_{D} is the radius of curvature of the droplet.

S.5.3.4 Ridge height

To calculate the height of the ridge, we calculate the density contour $\varrho_o(\rho, z) = \rho_o/2$ in the presence of a droplet. The apex of the ridge is selected as the point with the largest z-coordinate $z_{\max}$. The height of the ridge is then calculated as

$$h(t) = z_{\max}(t) - z_B(t) \tag{S.66}$$

where z_B is the z-coordinate of the oil in the brush far away from the droplet.

S.5.4 Calculating the thickness of the cloak

To quantify the thickness of the cloak with time, we use the relation

$$N = \int \varrho_o r^2 \sin\theta \, \mathrm{d}r \, \mathrm{d}\theta \, \mathrm{d}\varphi \tag{S.67}$$

where N is the number of oil monomers in the integration volume and $\varrho_o(r, \theta, \varphi)$ is the local density of oil in spherical coordinates centered at the center of curvature of the droplet. Since the cloak thickness is much smaller than the drop radius of curvature we set $r = R_\mathrm{D}$, and since we have azimuthal symmetry, we rewrite Eq. S.67 as

$$\begin{aligned} N(t) &= 2\pi R_\mathrm{D}^2 \int_{\theta_\mathrm{tip}(t)}^{\theta_\mathrm{ridge}} \int_0^\infty \varrho_o \sin\theta \, \mathrm{d}r \, \mathrm{d}\theta \\ &= 2\pi R_\mathrm{D}^2 \int_{\theta_\mathrm{tip}(t)}^{\theta_\mathrm{ridge}} \Sigma \sin\theta \, \mathrm{d}\theta. \end{aligned} \tag{S.68}$$

where θ_tip is the angular position of the cloak front and θ_ridge is the angular position of the wetting ridge, and we define $\Sigma = \int_0^\infty \varrho_o \, \mathrm{d}r$. The ridge remains more or less unchanged as the cloaking progresses; therefore, we determine θ_ridge manually at $\theta_\mathrm{ridge} = 65°$ from the $\Sigma(\theta)$ curve at the highest swelling ratio, and use this value for all lower swelling ratios. Given the number of oil monomers, the thickness of the cloak can be written as [51]

$$h = \frac{N}{\rho_o A} \tag{S.69}$$

with ρ_o and A the bulk density of oil and the area covered by the cloak respectively. The area can be calculated through

$$A(t) = 2\pi R_\mathrm{D}^2 (\cos\theta_\mathrm{tip}(t) - \cos\theta_\mathrm{ridge}) \tag{S.70}$$

Finally, using Eqs. (S.68), (S.69), and (S.70), we can calculate the thickness of the cloak from $\Sigma(\theta, t)$ as

$$h(t) = \frac{\int_{\theta_\mathrm{ridge}}^{\theta_\mathrm{tip}(t)} \Sigma \sin\theta \, \mathrm{d}\theta}{\rho_o \left[\cos\theta_{tip}(t) - \cos\theta_{ridge}\right]}. \tag{S.71}$$

S.5.5 Calculating the interaction function from simulations

To calculate the interaction function from Eq. S.14 we need to evaluate $\tilde{\mathcal{F}}_0(\varphi, \mu)$ which depends on the chemical potential in the brush. When our brush is at equilibrium, it coexists with a vapor phase of oil chains. This means that the chemical potential in the brush is equal to that of the vapor. Keeping in mind that our reference chemical potential is that of a saturated brush, we can find the chemical potential of an undersaturated brush through

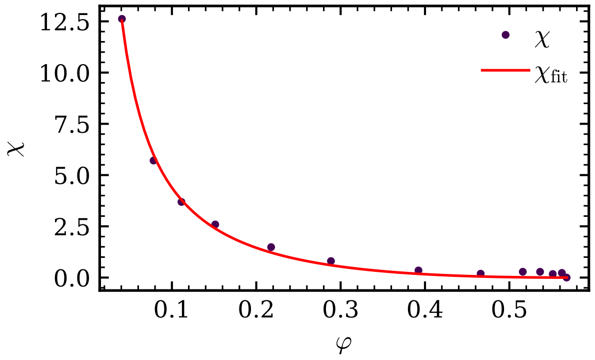

Fig. S.3: The swelling dependent interaction function $\chi(\varphi)$ as calculated from our simulation data. The red line is a fit to the function S.74.

$$\mu_B(\varphi) = k_B T \ln \frac{\rho_{\mathrm{v}}(\varphi)}{\rho_{\mathrm{v}}^{\mathrm{sat}}} \tag{S.72}$$

with $\rho_{\mathrm{v}}(\varphi)$ the density of the vapor of polymers, $\rho_{\mathrm{v}}^{\mathrm{sat}}$ the vapor density for a saturated brush. We then use Eq. S.14 to calculate $\chi(\varphi)$. For the fraction of oil φ we use the definition

$$\varphi = 1 - \frac{1}{\alpha} \tag{S.73}$$

where $\alpha = H_B/H_0$ is the swelling ratio of the brush. The resulting $\chi(\varphi)$ is shown in Figure S.3. To implement the interaction function in our diffusion model we choose to fit the simulation data to an ad-hoc function of the form

$$\chi(\varphi) = A \frac{(\varphi^* - \varphi)^2}{\varphi^\beta}. \tag{S.74}$$

After fitting, we find the values for the parameters

$$A = 2.48 \pm 0.28\,;\ \beta = 0.90 \pm 0.04 \tag{S.75}$$

where the errors are obtained from the covariance matrix of the fit.
While the fit is not perfect, it provides a good starting point to see what a variable χ model can achieve with the diffusion equation. In the diffusion model, some derivatives of the interaction function appear, which we write through our fit function as

$$\chi'(\varphi) = -\frac{\beta}{\varphi}\chi - 2\frac{A}{\varphi^\beta}(\varphi^* - \varphi) \tag{S.76}$$

$$\chi''(\varphi) = -\frac{\beta}{\varphi}\chi' + \frac{\beta}{\varphi^2}\chi + 2\beta\frac{A}{\varphi^{\beta+1}}(\varphi^* - \varphi) + 2\frac{A}{\varphi^\beta} \tag{S.77}$$

$$\chi^{(3)}(\varphi) = -\frac{\beta}{\varphi}\chi'' + 2\frac{\beta}{\varphi^2}\chi' - 2\frac{\beta}{\varphi^3}\chi - 2\beta(\beta+1)\frac{A}{\varphi^{\beta+2}}(\varphi^* - \varphi) - 4\beta\frac{A}{\varphi^{\beta+1}} \tag{S.78}$$

S.5.6 Determining continuum model parameters

In our dynamical equations above, we have many undetermined parameters, some of which we introduce heuristically such as the saturation line density λ_0 or the brush elastic constant k. Many of our model parameters can be determined from the results of our simulations, as described in this section.

S.5.6.1 Saturation fraction

To determine the saturation fraction, we measure the height of the brush H_B from the simulations, from that we calculate the fraction of oil as

$$\varphi = 1 - \frac{H_0}{H_B}. \tag{S.79}$$

When the brush is oversaturated with oil, the value calculated from the above equation will correspond to the saturation fraction φ^*. We find for PDMS-like brushes $\varphi^* \approx 0.57$ and for PLMA-like brushes we find $\varphi^* \approx 0.59$.

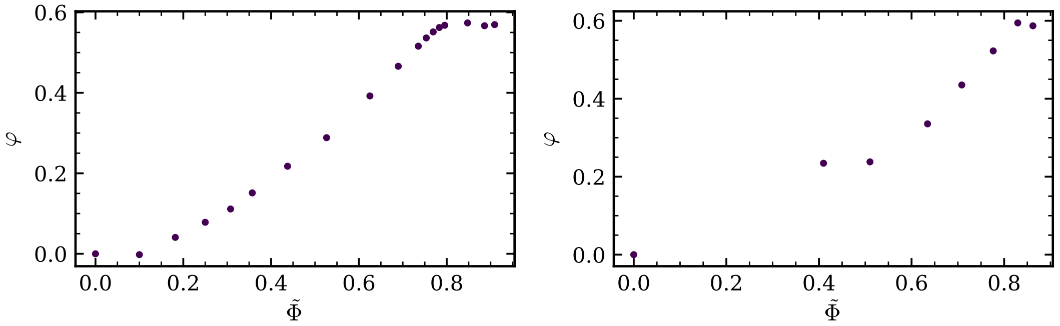

Fig. S.4: The lubricant fraction φ calculated from Eq. S.79 versus the number fraction of oil in the system $\tilde{\Phi}$ as calculated from Eq. S.58. The values at large φ correspond to the saturation fraction. (a) is the result for PDMS-like brushes with $\varphi^* \approx 0.57$, (b) for the PLMA-like brushes with $\varphi^* \approx 0.59$.

S.5.6.2 Elastic constant

According to Eq. S.14, the interaction part of the free energy vanishes when the brush is saturated at oil fraction φ^*. In addition, the chemical potential of the brush vanishes at saturation since we use the saturated brush as a reference. Therefore, the elastic constant of the brush can be determined by setting

$$\mu_B^{\mathrm{eq}}(\varphi^*) = 0 = \ln \varphi^* + 1 - \varphi^* + k \frac{\sigma^2 N_o}{1 - \varphi^*}. \tag{S.80}$$

Since φ^* can be determined from the simulation, the only unknown is k.

$$k = -\frac{1 - \varphi}{\sigma^2 N_o} \left(\ln \varphi^* + 1 - \varphi^* \right) \tag{S.81}$$

We find for PDMS-like brushes $k = 0.182$ and for PLMA-like brushes $k = 0.154$

S.5.6.3 Height of dry brush

The height of the dry brush can be easily extracted from the simulations with no oil. For PDMS-like brushes, one gets $H_0 = 9.431\,[r_c]$, and for PLMA-like brushes, $H_0 = 9.872\,[r_c]$.

S.5.6.4 Equilibrium line density

The value of λ_e can be determined from simulation by taking the total number of monomers in the ridge/cloak at equilibrium $N_{\rm cl}$, and dividing the result with the circumference of the contact line

$$\lambda_e = \frac{N_{\rm cl}}{2\pi R_{\rm cl}}. \tag{S.82}$$

In practice, we average the last 3 points in out $\lambda(t)$ time-series.

S.5.6.5 Contact line free energy density

The parameters λ_0 and κ can be calculated by fitting Eq. S.28 for different oil fractions. The value of λ_e can be calculated as described in this section, while the chemical potential can be calculated using Eq. S.20. The results are shown in Figure S.5 for variable interaction function in (a) and for $\chi = 0$ in (b). We find for PDMS-like brushes $\lambda_0 \approx 423.8$; $\kappa \approx 2.35 \times 10^{-3}$ for variable interaction function and $\lambda_0 \approx 430$; $\kappa \approx 1.02 \times 10^{-3}$ for $\chi = 0$.

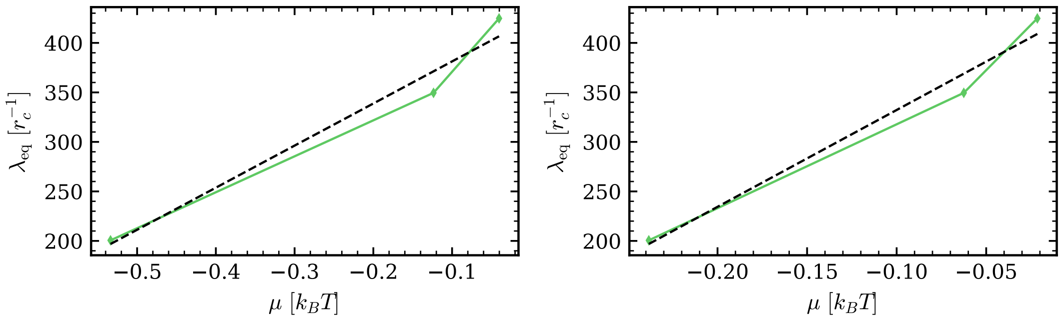

Fig. S.5: Equilibrium values for the line density λ_e versus the chemical potential in the brush calculated using (a) variable interaction function $\chi(\varphi)$ and (b) vanishing interaction function $\chi = 0$. The dashed lines are linear fits.

S.6 Additional Data on Oil Separation

In this section we present additional results for the separation of oil from the brush in the wetting ridges of the W-H system. Figure S.6 shows the density profile of grafted chains, oil chains, and the sum of the two. The insets show the full density map of polymers $\varrho_P(\rho, z)$ with the grafted chains labeled in red and the oil in blue. The magenta color indicates regions where the two are mixed, and the dashed lines show our choice of the position of the wetting ridge at $\rho = 35\,[r_c]$. We can clearly see that for the low swelling ratio there is not separation since the density of oil is always lower than the sum. Close to the saturation point, at $\alpha = 2.1$, the oil separates from the ridge. For the oversaturated brush, separation is also observed as expected.

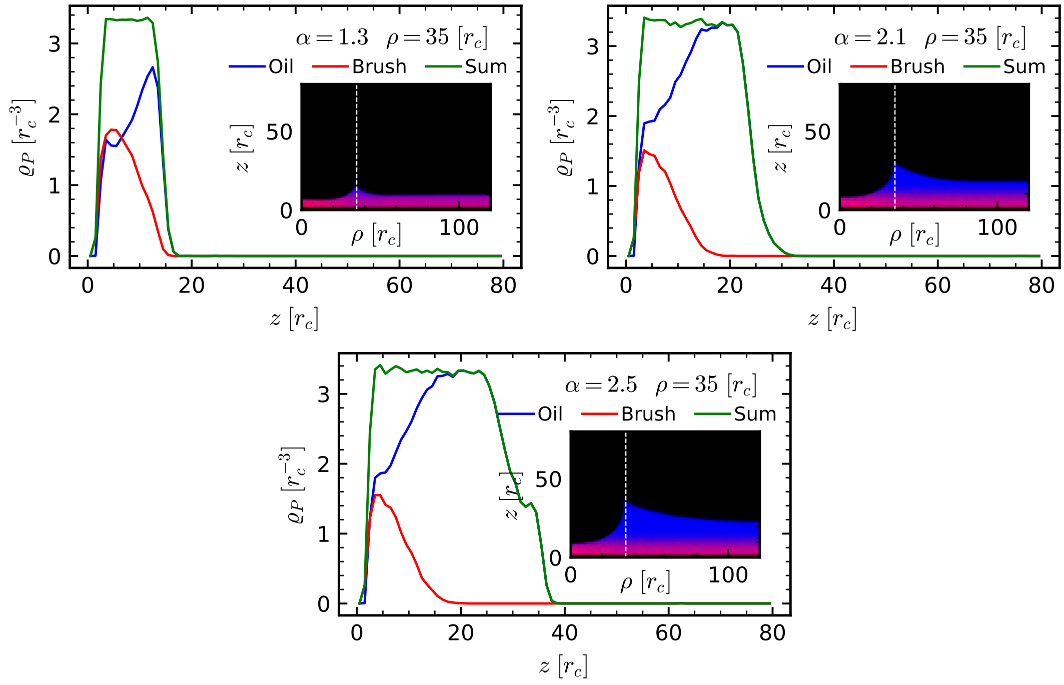

Fig. S.6: Vertical density profile of polymers in the W-H system in the area of the wetting ridge at radial position $\rho = 35\,[r_c]$ for swelling ratios (a) $\alpha = 1.3$, (b) $\alpha = 2.1$ and (c) $\alpha = 2.5$ (oversaturated). Insets show the density map $\varrho(\rho, z)$ with the grafted chains in red and the oil in blue. Magenta indicates a mixture of the two. The dashed lines indicate the position where the density profile is shown. For the higher swelling ratios we clearly see that the fluid separates from the brush as indicated by a density of oil equal to the bulk density of polymers.

To confirm that the fluid separation is due to the drop and localized in the wetting ridge, we also look at the density profiles of the grafted chains, oil chains, and the sum far away from the ridge at $\rho = 80\,[r_c]$. We only show the profiles for undersaturated brushes or close to saturation. The results are shown in Figure S.7 where it is clear that no separation occurs far away from the ridge.

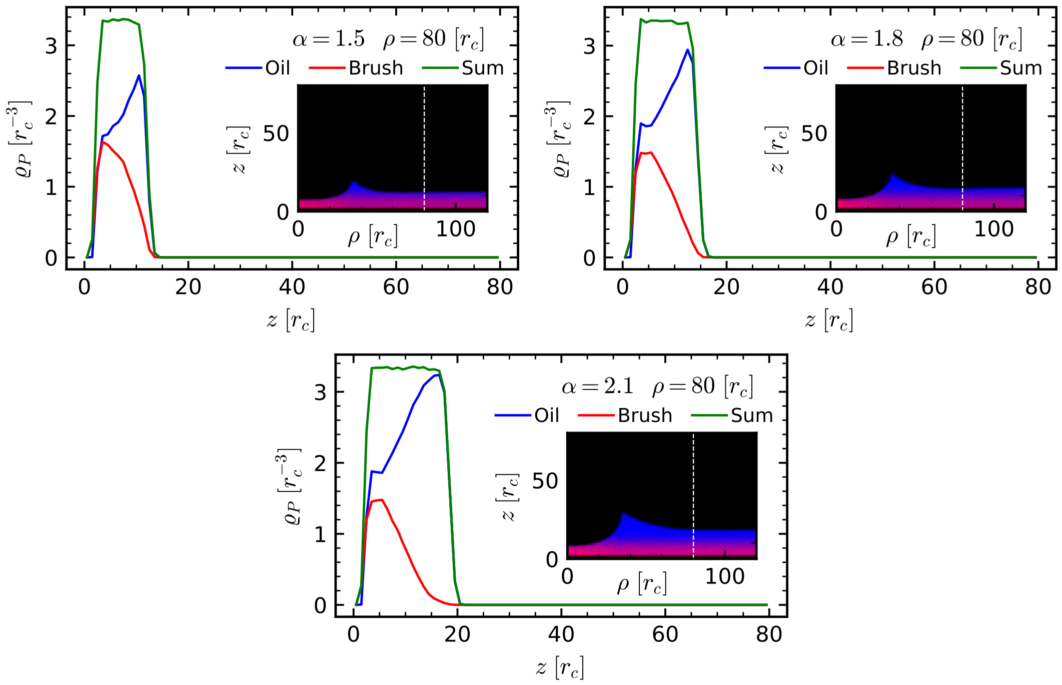

Fig. S.7: Similar to Fig. S.6 but at $\rho = 80\,[r_c]$ for swelling ratios (a) $\alpha = 1.5$, (b) $\alpha = 1.8$ and (c) $\alpha = 2.1$.

S.7 Contacting drops

The snapshots in Fig. S.8 show the final configurations after 6.4×10^3 $[t]$ in simulations of contacting droplets of the liquid and the oil for the three systems we investigated. We clearly see that the oil cloaks the liquid in the W-S and D-H systems, where the spreading parameter of oil on the liquid is positive. In the W-H system the configuration is that of two fused immiscible droplets.

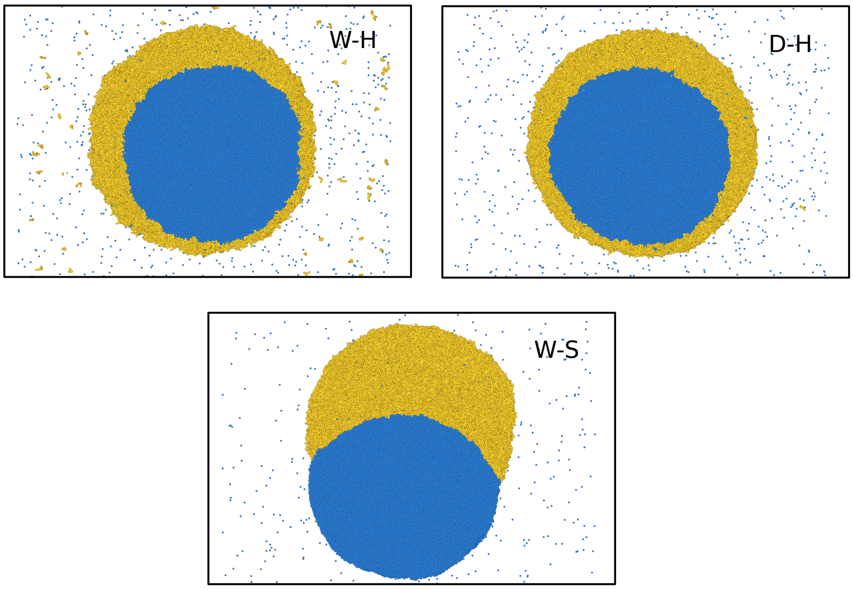

Fig. S.8: Snapshots from simulations of the systems analogous to water on PLMA, DMSO40 on PLMA, and water on PDMS. Yellow chains are oil, red are grafted chains, and blue particles constitute the liquid. The simulation time is $6.4 \times 10^3[\tau]$.

S.8 Oil distribution at lower swelling

Fig. S.9 shows the oil distribution during ridge growth at low swelling ratio $\alpha = 2$. The depletion zone is localized to the vicinity of the wetting ridge, as opposed to the case of higher swelling ratios, where it extends further.

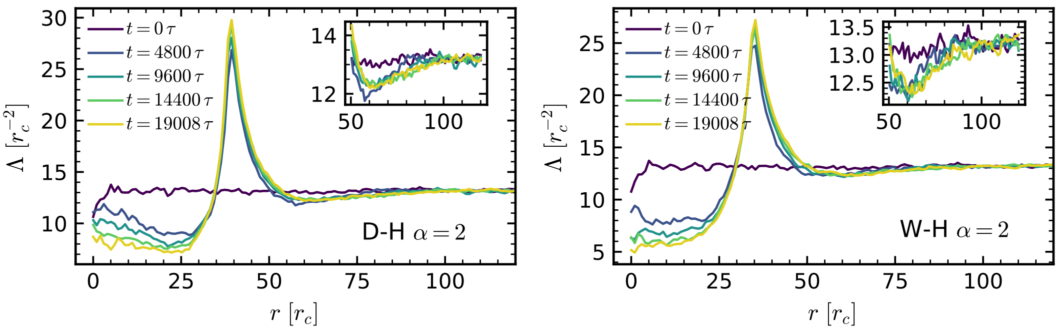

Fig. S.9: $\Lambda(\rho)$ at different time points during cloaking for the (a) D-H, (b) W-H, and (c) W-S systems at $\alpha = 2$. Insets are close ups near the three phase contact line. There is a clear depletion zone of material outside of the drop. The amount of material also drops from under the droplet.

S.9 Comparison of theoretical models

Fig. S.10 shows the simulation data for the line density at the three phase contact line along with the results of the diffusion model with variable interaction function $\chi(\varphi)$ or with vanishing interaction term $\chi = 0$.

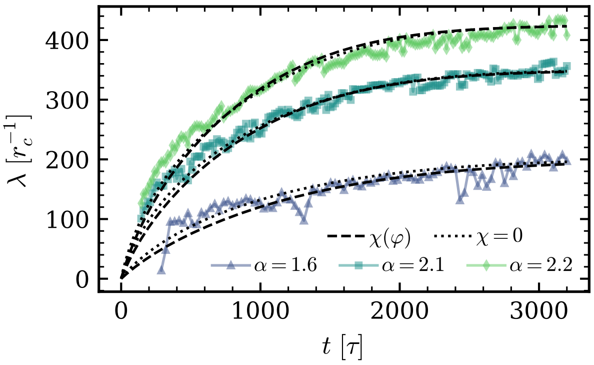

Fig. S.10: The line density at the ridge from simulation (data points), theory with variable interaction term (dashed lines), and theory with no interaction term (dotted lines).
